# Differential Association Between Ten Indices of Insulin Resistance and End-Organ Damage in a Community of African Ancestry in Africa

**DOI:** 10.3390/jcm14082703

**Published:** 2025-04-15

**Authors:** Angela J. Woodiwiss, Gavin R. Norton, Carlos D. Libhaber, Pinhas Sareli, Patrick H. C. Dessein

**Affiliations:** Cardiovascular Pathophysiology and Genomics Research Unit, Department of Physiology, School of Biomedical Sciences, Faculty of Health Sciences, University of the Witwatersrand, Johannesburg 2193, South Africapatrick.dessein22@gmail.com (P.H.C.D.)

**Keywords:** insulin resistance, cardiac function, renal function, vascular function

## Abstract

**Objective:** Various insulin resistance (IR) indices have been developed to assess cardiovascular (CVS) risk. We compared the association between ten IR indices and cardiac, renal, and vascular end-organ measures in a predominantly young (age 45.0 ± 18.3 years) South African Black population. **Methods:** We assessed the relationships between ten IR indices (homeostatic model assessment for IR [HOMA-IR], quantitative insulin sensitivity check index [QUICKI], metabolic score for IR [METS-IR], triglyceride–glucose index [TyG], TyG–body mass index [TyG-BMI], TyG–waist circumference [TyG-WC], TyG–waist-to-height ratio [TyG-WHtR], triglyceride to high-density cholesterol concentration [TyG-HDL], lipid accumulation product [LAP], visceral adiposity index [VAI]) and end-organ measures in 779 community participants of African ancestry. **Results:** HOMA-IR and QUICKI were the only IR indices consistently associated with end-organ measures (left ventricular [LV] mass index, *p* ≤ 0.005; LV relative wall thickness, *p* < 0.0001; early-to-late mitral velocity, *p* ≤ 0.01; E/e’, *p* ≤ 0.002; e’, *p* < 0.0001; pulse wave velocity, *p* = 0.036 (HOMA-IR only); glomerular filtration rate [GFR], *p* < 0.0001), independent of confounders. Furthermore, HOMA-IR was consistently higher, and QUICKI lower, in those with compared to those without end-organ damage (LV hypertrophy [*p* ≤ 0.03], concentric LV [*p* < 0.03], and reduced GFR [*p* ≤ 0.008]), independent of confounders. Importantly, the associations between HOMA-IR or QUICKI and end-organ measures were independent of additional CVS risk factors, including adiposity measures, and were replicated in the participants without diabetes mellitus (n = 669) and in the participants without high blood pressure (n = 505). **Conclusions:** In a predominantly young community of African ancestry, of ten recommended IR indices, only HOMA-IR and QUICKI were consistently associated with end-organ damage independent of CVS risk factors.

## 1. Introduction

The prevalence of obesity is escalating worldwide [1]. Consequently, the rate of obesity-associated insulin resistance (IR) is also increasing [2]. As IR exacerbates the risk of CVD [3], it is imperative to consider IR in CVS risk assessments. The gold standard assessment of IR, the hyperinsulinemic–euglycemic clamp technique, is complex, time-consuming, and, hence, not feasible in routine clinical practice. Therefore, the homeostasis model assessment of insulin resistance (HOMA-IR) is widely used in population studies [4]. However, HOMA-IR requires the laboratory assessment of fasting insulin concentrations and is costly; hence, a number of other indices of IR have been developed [5,6,7,8,9,10,11]. These alternative indices use various combinations of measures including body mass index (BMI), waist circumference, waist-to-height ratio, and plasma glucose, triglyceride, and high-density lipoprotein (HDL) cholesterol concentrations.

Recently, in a large follow-up study (NHANES 2001–2018) assessing four indices of IR, only the metabolic score for insulin resistance (METS-IR), but neither the triglyceride glucose index (TyG index), triglyceride–HDL-cholesterol ratio (TyG-HDL), nor HOMA-IR, was associated with all-cause and CVD mortality in the general population [12]. The associations between METS-IR and mortality were predominantly present in a nonelderly (aged < 65 years) population [12]. These data suggest that METS-IR is the preferable index of IR to predict all-cause and CVD mortality in young populations [12]. In contrast, in young (aged 35–64 years) individuals without diabetes mellitus (DM), the TyG index was preferable to HOMA-IR in predicting the risk of coronary heart disease (CHD) [13]. Moreover, the strength of the relationship between indices of IR and the risk of CHD was found to differ across populations [13]. Indeed, there is growing evidence that the impact of IR on cardiovascular health may differ by race, with a lesser impact in the Black population [14,15,16]. Notably, Black populations have higher IR but lower triglyceride and higher HDL cholesterol concentrations than White populations [17]. Despite the higher IR, the relationship between IR and CVD risk factors is weaker in Black compared to White populations [17]. Hence, indices of IR may underestimate cardiovascular risk in Black individuals [18].

Nevertheless, assessments of IR are essential in African populations to mitigate high CVD mortality rates. In this regard, there is rising concern regarding the probability of a pandemic of CVD in Sub-Saharan Africa [19], which is compounded by the current high burden of obesity in these countries [20]. A further mitigating factor is the earlier onset (~15 years) of cardiovascular outcomes in people of African compared to European descent [21,22]. Hence, the efficacy of various indices of IR in assessing cardiovascular risk in people of African ancestry, especially young adults, requires investigation. We, therefore, aimed to compare the association between various indices of IR and measures of cardiac, renal, and vascular function (end-organ measures) in a predominantly young (83% < 65 years of age) and overweight (25%) or obese (41%) South African Black population.

## 2. Methods

### 2.1. Study Group

The present study was conducted according to the guidelines of the Declaration of Helsinki and approved by the Human Research Ethics Committee (Medical) of the University of the Witwatersrand (protocol number: M02-04-72 and renewed as M07-04-69, M12-04-108, M17-04-01, and M22-03-93; approved on 4 April 2022). The participants gave informed written consent. The design of this study was previously described [23,24]. Briefly, families of black African descent (Nguni and Sotho chiefdoms) with siblings older than 16 years of age were randomly recruited from the South West Township (SOWETO) of Johannesburg, South Africa. The current study is a sub-study of the large cross-sectional community-based study in adults, conducted in a laboratory setting. The inclusion criteria for this sub-study were (1) the presence of the data required for the calculation of IR indices (fasting insulin, blood glucose, triglyceride, and high-density lipoprotein cholesterol concentrations, body mass index, waist circumference, and waist-to-height ratio) and (2) the presence of measures of cardiac (LVMI, LV RWT, mFS, EF, E/A), renal (GFR), and vascular (PWV) function. Further assessments of cardiac diastolic function (E/e’ and e’) were only performed in 516 of the 779 included participants. As the presence of diabetes mellitus (DM) may impact the relationships between indices of IR and end-organ measures, sensitivity analysis was also conducted in the subgroup of participants who did not have DM (n = 669 and n = 444 with E/e’ and e’). As the end-organ measures assessed are closely linked to hypertension, sensitivity analyses were also conducted in the participants who did not have hypertension (normotensive participants [NT]) plus the hypertensive (HT) participants with controlled blood pressure (NT + HT with controlled BP, n = 505). The sample sizes are clarified in Figure S1.

### 2.2. Clinical, Demographic, Anthropometric, and Laboratory Information

Demographic and clinical data were obtained by means of a questionnaire [23]. Standard approaches were used to measure height, weight, and waist circumference. The participants were considered to be overweight if their body mass index (BMI) was ≥25 kg/m^2^ and obese if their BMI was ≥30 kg/m^2^. Blood glucose, lipid profiles, and percentage glycated haemoglobin (HbA1c) were assessed after at least a 12 h fast. Diabetes mellitus (DM) was defined as an HbA1c value greater than 6.5% or the use of insulin or oral-glucose-lowering agents. Increased LDL-cholesterol was defined as LDL-cholesterol ≥ 3.0 mmol/L, increased triglycerides was defined as ≥1.7 mmol/L, and decreased HDL-cholesterol was defined as ≤1 mmol/L for men and ≤1.3 mmol/L for women. High-quality office brachial blood pressure (BP) measurements were obtained according to guidelines, after 5 min of rest in the seated position, by a trained nurse-technician using a standard mercury sphygmomanometer [23]. Office brachial BP was the mean of 5 measurements obtained at least 30 s apart. Raised BP was defined as a mean office brachial systolic BP ≥ 140 mm Hg or diastolic BP ≥ 90 mm Hg based on an average of five consecutive measurements on one occasion [12]. Hypertension was based on a raised BP or the use of antihypertensive medications. Normotension was defined as having a systolic BP < 140 mm Hg and diastolic BP < 90 mm Hg whilst not receiving any antihypertensive medication. Controlled BP was defined as systolic BP < 140 mm Hg and diastolic BP < 90 mm Hg whilst receiving antihypertensive medication.

### 2.3. Insulin and Indices of Insulin Resistance

Fasting plasma insulin concentrations were determined from an insulin immulite, solid-phase, two-site chemiluminescent immunometric assay (Diagnostic Products Corporation, Los Angeles, CA, USA). Ten indices of IR were calculated as follows:

1. Homeostasis model assessment of insulin resistance (HOMA-IR) = (insulin [µU/mL] × glucose [mmol/L])/22.5.
2. Quantitative insulin sensitivity check index (QUICKI) = 1/(log insulin[µU/mL] + log glucose [mg/dL]) [25].
3. Metabolic score for insulin resistance (METS-IR) = (ln((2 × glucose [mg/dL]) + triglycerides [mg/dL])) × (BMI/(ln(HDL-cholesterol [mg/dL]))) [5].
4. Triglyceride–glucose index (TyG) = ln(triglycerides [mg/dL] × glucose [mg/dL]/2) [6].
5. Triglyceride–body mass index (TyG-BMI) = TyG × BMI [7].
6. Triglyceride–waist circumference (TyG-WC) = TyG × waist circumference [7].
7. Triglyceride–waist-to-height ratio (TyG-WHtR) = TyG × waist-to-height ratio [8].
8. Triglyceride to high-density cholesterol concentration (TyG-HDL) = triglycerides [mg/dL]/HDL-cholesterol [mg/dL] [9].
9. Lipid accumulation product (LAP) = (waist circumference − 65) × triglycerides [mmol/L] for men and LAP = (waist circumference − 58) × triglycerides [mmol/L] for women [10].
10. Visceral adiposity index (VAI) = (waist circumference/(39.68 + (1.88 × BMI))) × (triglycerides [mmol/L]/1.03) × (1.31/HDL-cholesterol [mmol/L]) for men and VAI = (waist circumference/(36.58 + (1.89 × BMI))) × (triglycerides [mmol/L]/0.81) × (1.52/HDL-cholesterol [mmol/L]) for women [11].

### 2.4. End-Organ Measures

Left ventricular geometry and function were assessed using echocardiography (Acuson SC2000 Diagnostic ultrasound system, Siemens Medical Solutions USA, Inc., Malvern, PA, USA). Echocardiographic measurements were recorded and analysed off-line by experienced investigators (CDL and AJW) who were unaware of the clinical data of the participants and who had a low degree of inter- and intra-observer variability [23]. The left ventricular mass index (LVMI) was determined from transthoracic two-dimensional targeted M-mode echocardiography obtained in the long-axis parasternal view. The variables were analysed according to the American Society of Echocardiography convention [26]. Left ventricular mass was determined using a standard formula [27] and indexed to body surface area (LVMI) [24]. Left ventricular hypertrophy was defined as LVMI ≥ 95 g/m^2^ in women and ≥115 g/m^2^ in men. Left ventricular relative wall thickness (LV RWT) was calculated as (LV anterior + posterior wall thickness at end diastole)/LV end diastolic diameter. Concentric remodelling was defined as LV RWT ≥ 0.42. Left ventricular systolic function was assessed from midwall fractional shortening (mFS) using a standard M-mode approach and from ejection fraction (EF) using Simpson’s biplane method. Early-to-late transmitral blood flow velocity (E/A) and myocardial tissue lengthening in early diastole at the mitral annulus (e’) were determined in the 4-chamber apical view. The E/e’ ratio (an index of LV filling pressures) was calculated.

Glomerular filtration rate (GFR) was determined using the 2021 Chronic Kidney Disease Epidemiology Collaboration (CKD-EPI) equations [28]:

Women with serum creatinine (SCr) ≤ 0.7 mg/dL: GFR = 142 × (SCr/0.7)^−0.241^ × (0.9938)^Age^ × 1.012.
Women with SCr > 0.7 mg/dL: GFR = 142 × (SCr/0.7)^−1.200^ × (0.9938)^Age^ × 1.012.
Men with SCr ≤ 0.9 mg/dL: GFR = 142 × (SCr/0.9)^−0.302^ × (0.9938)^Age^.
Men with SCr > 0.9 mg/dL: GFR = 142 × (SCr/0.9)^−1.200^ × (0.9938)^Age^.
Decreased GFR was identified as GFR less than 90 mL/min per 1.73 m^2^.

As an index of vascular function (aortic stiffness), carotid–femoral (aortic) pulse wave velocity (PWV) was determined from sequential waveform measurements at carotid and femoral sites using applanation tonometry and SphygmoCor version 9.0 software (AtCor by CARDIEX Limited, Sydney, New South Wales, Australia) as previously described [23]. After the participants had rested for 15 min in the supine position, arterial waveforms at the carotid and femoral pulse were sequentially recorded by applanation tonometry during an 8 s period using a high-fidelity SPC301 micromanometer (Millar Instrument, Inc., Houston, TX, USA). We discarded recordings where the systolic or diastolic variability in consecutive waveforms exceeded 5% or the amplitude of the pulse wave signal was less than 80 mV. Distances from the suprasternal notch to the carotid sampling site (distance A) and from the suprasternal notch to the femoral artery (distance B) were measured. Pulse wave velocity distance was calculated as distance B minus distance A. The time delay in the pulse waves between the carotid and femoral sites was determined using an electrocardiograph-derived R wave as a fiducial point. Pulse transit time was the average of 10 consecutive beats. Aortic PWV was calculated as the ratio of the distance in meters to the transit time in seconds.

### 2.5. Data Analysis

Database management and statistical analyses were performed with SAS software, version 9.4 (The SAS Institute, Cary, NC, USA). Continuous variables are expressed as mean (±SD) for parametric data or mean (interquartile range) for non-parametric data. Dichotomous variables are expressed as percentages. As HOMA-IR, Tyg-HDL, LAP, and VAI were not normally distributed, they were logarithmically transformed (natural logarithm, ln) prior to performing linear regression analyses. To identify independent relationships, multivariate-adjusted linear regression analysis was performed. Adjustments were for age, sex, regular alcohol intake, regular tobacco intake, heart rate, treatment for hypertension, and brachial pulse pressure (PP). To determine whether the associations between HOMA-IR or QUICKI (dependent variable) and end-organ damage were independent of additional CVS risk factors, further adjustments were made for triglycerides, HDL-cholesterol, waist circumference (or BMI), SBP, and DBP (instead of PP). Logistic regression analysis was performed to determine the odds of end-organ damage in association with one SD increment in HOMA-IR or QUICKI, independent of confounders. As the presence of DM may affect relationships between indices of IR and end-organ measures, sensitivity analysis was conducted on the participants who did not have DM. As the end-organ measures assessed are closely linked to hypertension, sensitivity analyses were also conducted on the participants who did not have hypertension (normotensive participants [NT]) plus hypertensive participants with controlled blood pressure (NT + HT with controlled BP). As more women than men volunteered for this study, sensitivity analysis was also conducted on women and men separately.

## 3. Results

### 3.1. Participant Characteristics

Table 1 shows the general characteristics of all the participants as well as the participants without DM. The majority of the participants were young (mean age 45.0 ± 18.3 years, with >80% being <65 years of age). A high proportion of the participants had hypertension (>40%). A significant proportion of the participants had uncontrolled BP (all: 35.2%; without DM: 32.6%). Of those participants with hypertension, BP was controlled in only 24.7% (without DM: 18.7%). Approximately half of those participants with hypertension were receiving antihypertensive medication (all: 56.9%; without DM: 48.9%). A high proportion of the participants were overweight (>25%) or obese (>37%), 14.1% of all the participants had DM, and a high proportion of those without DM had pre-DM (55.1%, based on HbA1c ≥ 5.7). Only a third of the participants had elevated LDL cholesterol concentrations (all: 34.7%; without DM: 31.4%), few had elevated triglycerides (all: 16.6%. without DM: 12.9%), and only about a quarter had decreased HDL cholesterol concentrations (all: 28.9%; without DM: 25.9%). None of the participants were receiving lipid-lowering therapy. None of the participants had EF < 40%, and only eight (1.0%) of the participants had an LVEDD ≥ 5.5 cm. In these eight participants, the mean EF was 46.4 ± 2.2%, the mean age was 61.2 ± 12.9 years, and the mean E/e’ was 7.07 ± 2.80. Hence, it appears highly unlikely that any of the participants had dilated cardiomyopathy. Furthermore, only six (0.77%) of the participants had either LV posterior (PWED) or septal (SEPED) wall thickness ≥ 15 mm. All six of these participants had hypertension (mean SBP: 156 ± 15 mm Hg; mean DBP: 93 ± 9 mm Hg), and only two of them (33.3%) were receiving antihypertensive medication. The increased wall thickness in these participants (mean PWED: 14.9 ± 1.5 mm; mean SEPED: 16.9 ± 1.3 mm) was, therefore, most likely due to poorly managed high blood pressure and not hypertrophic cardiomyopathy. In general, the characteristics of the subgroup of the participants without DM were similar to those of all the participants. However, fasting plasma glucose concentrations, HbA1c, 6 of the 10 indices of IR (METS-IR, TyG, TyG-BMI, TyG-WC, TyG-WHtR, and LAP), and pulse wave velocity were lower, and QUICKI, e’, and GFR were higher, in the participants without DM compared to all the participants (Table 1). The subgroup of NT participants plus HT participants with controlled BP (Table S1) were younger, were less obese, and had lower BP; in this subgroup, 6 of the 10 indices of IR (METS-IR, TyG, TyG-BMI, TyG-WC, TyG-WHtR, and LAP), LVMI, LV RWT, E/e’, and PWV were lower and mFS, E/A, e’ and GFR were higher compared to all the participants.

### 3.2. Correlations Among Indices of Insulin Resistance

The heat map (Table 2) shows the Pearson’s correlations between the ten indices of IR. All ten indices of IR were significantly (*p* < 0.0001) correlated with each other. HOMA-IR and QUICKI were strongly inversely correlated with each other. However, HOMA-IR (positively) and QUICKI (negatively) were weakly correlated with the other eight indices of IR. The other eight indices of IR were strongly positively correlated with each other. The strongest correlations were between TyG-WC and TyG-WhtR (0.974) and between METS-IR and TyG-BMI (r = 0.965). The weakest correlations were between TyG-BMI and TyG-HDL (0.437) and between METS-IR and TyG (r = 0.498).

### 3.3. Unadjusted Associations Between Indices of Insulin Resistance and End-Organ Measures

Prior to any adjustments, all the indices of IR (HOMA-IR, QUICKI, METS-IR, TyG, TyG-BMI, TyG-WC, TyG-WHtR, TyG-HDL, LAP, and VAI) were associated with E/e’ and e’ (Table 3). All the indices of IR were associated with LVMI (except for VAI), LV RWT, E/A, PWV, and GFR (Table 3). None of the indices of IR were associated with EF or mFS (Table 3). Similarly, in the participants without DM (Table S2), prior to any adjustments, HOMA-IR, QUICKI, TyG, TyG-WC, TyG-WHtR, TyG-HDL, and LAP were associated with LVMI, LV RWT, E/A, E/e’, e’, PWV, and GFR, but not with EF or mFS. QUICKI was not associated with PWV, and the associations between METS-IR and LVMI and between TyG-BMI and LVMI did not achieve statistical significance in the cohort of participants without DM (Table S2). However, Pearson’s correlation coefficients were not dissimilar from those of all the participants. In the participants without DM, similar to in all the participants, VAI was associated with LV RWT, E/A, E/e’, e’, PWV, and GFR but not with LVMI, EF, or mFS (Table S2). In the normotensive participants plus the hypertensive participants with controlled BP (Table S3), prior to any adjustments, the associations between the IR indices and end-organ measures were similar to those noted in all the participants, except only HOMA-IR and QUICKI were associated with LVMI, and only HOMA-IR, QUICKI, TyG, and TYG-WHtR were associated with LV RWT. In the women, similar to all the participants, prior to any adjustments, all the indices of IR (HOMA-IR, QUICKI, METS-IR, TyG, TyG-BMI, TyG-WC, TyG-WHtR, and LAP), except for TyG-HDL and VAI, were associated with LVMI, LV RWT, E/A, E/e’, e’, PWV, and GFR (Table S4). None of the indices of IR were associated with EF or mFS (Table S4). TyG-HDL and VAI were associated with LV RWT, E/A, E/e’, e’, PWV, and GFR but not LVMI (Table S4). In the men, prior to any adjustments, most of the indices of IR (HOMA-IR, QUICKI, METS-IR, TyG-BMI, TyG-WC, TyG-WHtR, and LAP) were associated with LVMI, LV RWT, E/A, E/e’, e’, PWV, and GFR (Table S5). The associations between QUICKI and LVMI and between HOMA-IR or QUICKI and PWV did not achieve statistical significance in the men (Table S5). However, Pearson’s correlation coefficients were not dissimilar from those of the participants without DM. TyG, TyG-HDL, and VAI were associated with LV RWT, E/A, E/e’, e’, PWV, and GFR but not LVMI (Table S5). Only TyG-WC and TyG-WHtR were weakly associated with mFS (Table S5).

### 3.4. Adjusted Associations Between Insulin and Blood Glucose and End-Organ Measures

Table S6 shows the adjusted relationships between end-organ measures and insulin or blood glucose (BG), either separately or in the same model (n = 779). The relationships between HOMA-IR and end-organ measures are also shown for comparison. In the separate models, insulin was associated with LVMI, LV RWT, E/A, E/e’, e’, and GFR but not with PWV. Blood glucose was associated with LV RWT, E/e’, e’, GFR (trend, *p* = 0.0578), and PWV but not with LVMI or E/A. When assessed in the same model, both insulin and blood glucose were associated with LV RWT, E/A, and e’. In the same model, only insulin remained associated with LVMI, E/e’, and GFR, and only blood glucose remained associated with PWV.

### 3.5. Adjusted Associations Between Indices of Insulin Resistance and End-Organ Measures

**Figure 1:**
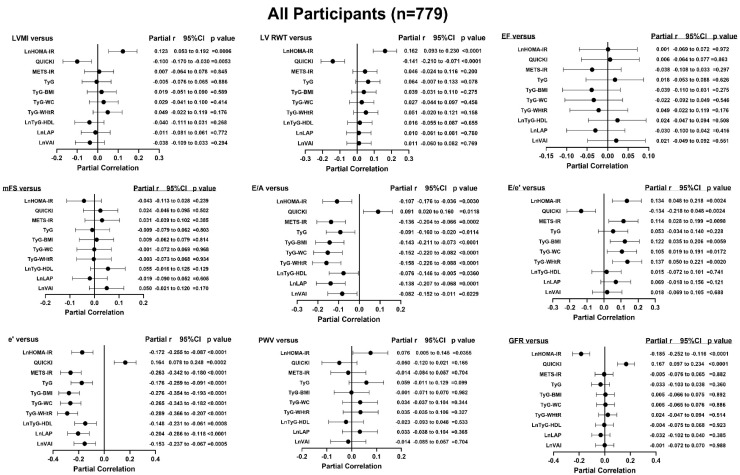
Relationships between various indices of insulin resistance and end-organ measures independent of confounders in all the participants (n = 779). Confounders are age, sex, regular alcohol intake, regular tobacco intake, treatment for hypertension, heart rate, and brachial pulse pressure. BMI, body mass index; E/A, early-to-late mitral velocity; e’, myocardial tissue lengthening in early diastole at the mitral annulus; E/e’, transmitral early blood flow velocity/velocity of the mean value of lateral and septal wall myocardial tissue lengthening in early diastole at the mitral annulus; EF, ejection fraction; GFR, estimated glomerular filtration rate; HDL, high-density lipoprotein; lnHOMA-IR, natural logarithm of homeostatic model assessment for insulin resistance; lnLAP, natural logarithm of lipid accumulation product; LDL, low-density lipoprotein; LVMI, left ventricular mass indexed to body surface area; LV RWT, left ventricular relative wall thickness; METS-IR, metabolic score for insulin resistance; mFS, left ventricular midwall fractional shortening; PWV, pulse wave velocity; QUICKI, quantitative insulin sensitivity check index; r, correlation coefficient; TyG, triglyceride–glucose index; TyG-BMI, triglyceride–body mass index; lnTyG-HDL, natural logarithm of triglycerides to high-density cholesterol concentrations; TyG-WC, triglyceride–waist circumference; TyG-WHtR, triglyceride–waist-to-height ratio; lnVAI, natural logarithm of visceral adiposity index.

**Figure 2:**
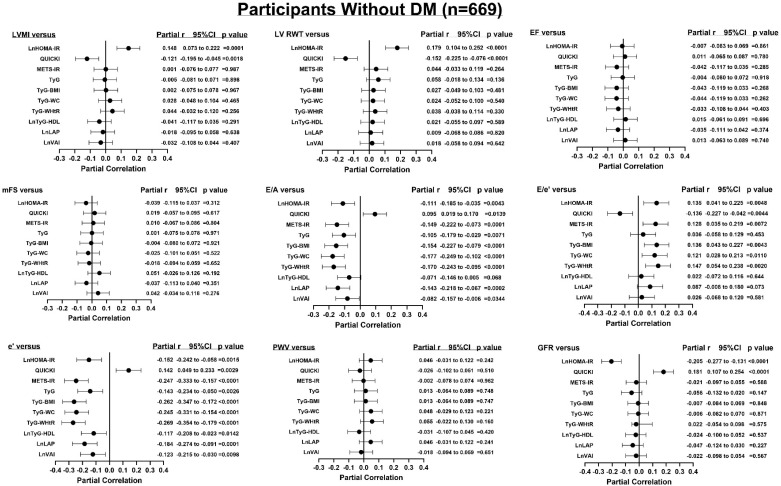
Relationships between various indices of insulin resistance and end-organ measures independent of confounders in the participants without DM (n = 669). Confounders are age, sex, regular alcohol intake, regular tobacco intake, treatment for hypertension, heart rate, and brachial pulse pressure. BMI, body mass index; E/A, early-to-late mitral velocity; e’, myocardial tissue lengthening in early diastole at the mitral annulus; E/e’, transmitral early blood flow velocity/velocity of the mean value of lateral and septal wall myocardial tissue lengthening in early diastole at the mitral annulus; EF, ejection fraction; GFR, estimated glomerular filtration rate; HDL, high-density lipoprotein; lnHOMA-IR, natural logarithm of homeostatic model assessment for insulin resistance; lnLAP, natural logarithm of lipid accumulation product; LDL, low-density lipoprotein; LVMI, left ventricular mass indexed to body surface area; LV RWT, left ventricular relative wall thickness; METS-IR, metabolic score for insulin resistance; mFS, left ventricular midwall fractional shortening; PWV, pulse wave velocity; QUICKI, quantitative insulin sensitivity check index; r, correlation coefficient; TyG, triglyceride–glucose index; TyG-BMI, triglyceride–body mass index; lnTyG-HDL, natural logarithm of triglycerides to high-density cholesterol concentrations; TyG-WC, triglyceride–waist circumference; TyG-WHtR, triglyceride–waist-to-height ratio; lnVAI, natural logarithm of visceral adiposity index.

**Figure 3:**
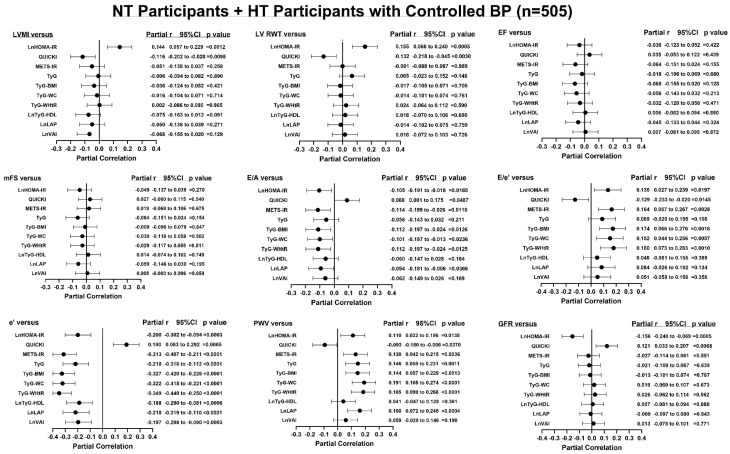
Relationships between various indices of insulin resistance and end-organ measures independent of confounders in the normotensive (NT) participants plus the hypertensive (HT) participants with controlled blood pressure (BP) (n = 505). Confounders are age, sex, regular alcohol intake, regular tobacco intake, treatment for hypertension, heart rate, and brachial pulse pressure. BMI, body mass index; E/A, early-to-late mitral velocity; e’, myocardial tissue lengthening in early diastole at the mitral annulus; E/e’, transmitral early blood flow velocity/velocity of the mean value of lateral and septal wall myocardial tissue lengthening in early diastole at the mitral annulus; EF, ejection fraction; GFR, estimated glomerular filtration rate; HDL, high-density lipoprotein; lnHOMA-IR, natural logarithm of homeostatic model assessment for insulin resistance; lnLAP, natural logarithm of lipid accumulation product; LDL, low-density lipoprotein; LVMI, left ventricular mass indexed to body surface area; LV RWT, left ventricular relative wall thickness; METS-IR, metabolic score for insulin resistance; mFS, left ventricular midwall fractional shortening; PWV, pulse wave velocity; QUICKI, quantitative insulin sensitivity check index; r, correlation coefficient; TyG, triglyceride–glucose index; TyG-BMI, triglyceride–body mass index; lnTyG-HDL, natural logarithm of triglycerides to high-density cholesterol concentrations; TyG-WC, triglyceride–waist circumference; TyG-WHtR, triglyceride–waist-to-height ratio; lnVAI, natural logarithm of visceral adiposity index.

After adjustments for confounders (age, sex, regular smoking, regular alcohol, treatment for hypertension, heart rate, brachial pulse pressure), only HOMA-IR was consistently associated with the end-organ measures (LVMI, LV RWT, E/A, E/e’, e’, PWV, and GFR) (Figure 1). QUICKI was associated with LVMI, LV RWT, E/A, E/e’, e’, and GFR but not with PWV (Figure 1). None of the other indices of IR were associated with LVMI, LV RWT, PWV, or GFR, independent of confounders (Figure 1). However, all ten of the indices of IR remained associated with E/A and e’ after adjustments for confounders. Similarly, in the participants without DM (Figure 2), only HOMA-IR and QUICKI were consistently associated with the end-organ measures (LVMI, LV RWT, E/A, E/e’, e’, and GFR). In the participants without DM, all ten of the indices of IR remained associated with E/A and e’ after adjustments for confounders (Figure 2). In the normotensive participants and the hypertensive participants with controlled BP (Figure 3), similar to in all the participants, only HOMA-IR and QUICKI were consistently associated with end-organ measures (LVMI, LV RWT, E/A, E/e’, e’, PWV, and GFR). In the women, as with all the participants, only HOMA-IR and QUICKI were consistently associated with the end-organ measures (LVMI, LV RWT, E/A, E/e’, e’, PWV, and GFR) independent of confounders (Table S7). In the men, HOMA-IR and QUICKI were associated with LVMI, LV RWT, E/e’, e’, and GFR but not with E/A or PWV, independent of confounders (Table S8). In addition, in the men, METS-IR was weakly associated with LV RWT, E/e’, e’, and GFR; Tyg-BMI with LV RWT, E/A, E/e’, e’, and GFR; Tyg-WHtR with LV RWT, E/A, E/e’, and e’; Tyg-WC with E/A, E/e’, and e’; and TyG with e’, independent of confounders (Table S8).

### 3.6. Associations Between Indices of Insulin Resistance and End-Organ Damage

**Figure 4:**
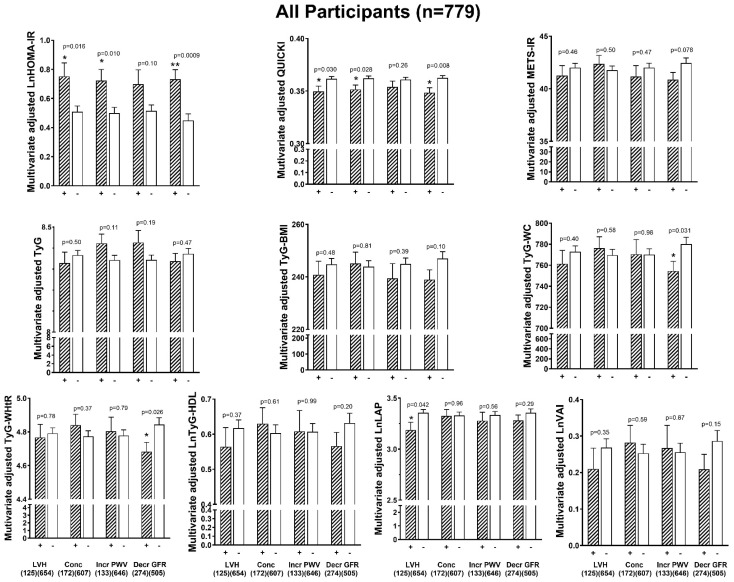
Adjusted mean values (±SEM) of the various indices of insulin resistance in those with (+) compared to those without (-) end-organ damage (adjustments are for age, sex, regular alcohol intake, regular tobacco intake, treatment for hypertension, heart rate, and brachial pulse pressure) in all the participants (n = 779). BMI, body mass index; Concentricity, increased left ventricular relative wall thickness; Decr, decreased; GFR, estimated glomerular filtration rate; HDL, high-density lipoprotein; Incr, increased; lnHOMA-IR, natural logarithm of homeostatic model assessment for insulin resistance; lnLAP, natural logarithm of lipid accumulation product; LDL, low-density lipoprotein; LVH-BSA, increased left ventricular mass indexed to body surface area; METS-IR, metabolic score for insulin resistance; PWV, pulse wave velocity; QUICKI, quantitative insulin sensitivity check index; TyG, triglyceride–glucose index; TyG-BMI, triglyceride–body mass index; lnTyG-HDL, natural logarithm of triglycerides to high-density cholesterol concentrations; TyG-WC, triglyceride–waist circumference; TyG-WHtR, triglyceride–waist-to-height ratio; lnVAI, natural logarithm of visceral adiposity index. * *p* < 0.05 and ** *p* < 0.001.

**Figure 5:**
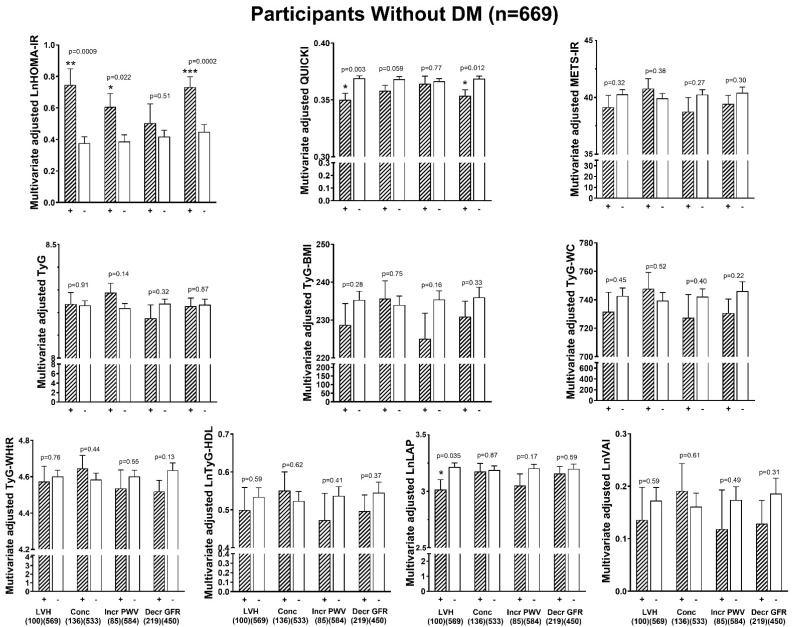
Adjusted mean values (±SEM) of various indices of insulin resistance in those with (+) compared to those without (-) end-organ damage (adjustments are for age, sex, regular alcohol intake, regular tobacco intake, treatment for hypertension, heart rate, brachial pulse pressure) in participants without DM (n = 669). BMI, body mass index; Concentricity, increased left ventricular relative wall thickness; Decr, decreased; GFR, estimated glomerular filtration rate; HDL, high-density lipoprotein; Incr, increased; lnHOMA-IR, natural logarithm of homeostatic model assessment for insulin resistance; lnLAP, natural logarithm of lipid accumulation product; LDL, low-density lipoprotein; LVH-BSA, increased left ventricular mass indexed to body surface area; METS-IR, metabolic score for insulin resistance; PWV, pulse wave velocity; QUICKI, quantitative insulin sensitivity check index; TyG, triglyceride–glucose index; TyG-BMI, triglyceride–body mass index; lnTyG-HDL, natural logarithm of triglycerides to high-density cholesterol concentrations; TyG-WC, triglyceride–waist circumference; TyG-WHtR, triglyceride–waist-to-height ratio; lnVAI, natural logarithm of visceral adiposity index. * *p* < 0.05, ** *p* < 0.001, and *** *p* < 0.0005.

**Figure 6:**
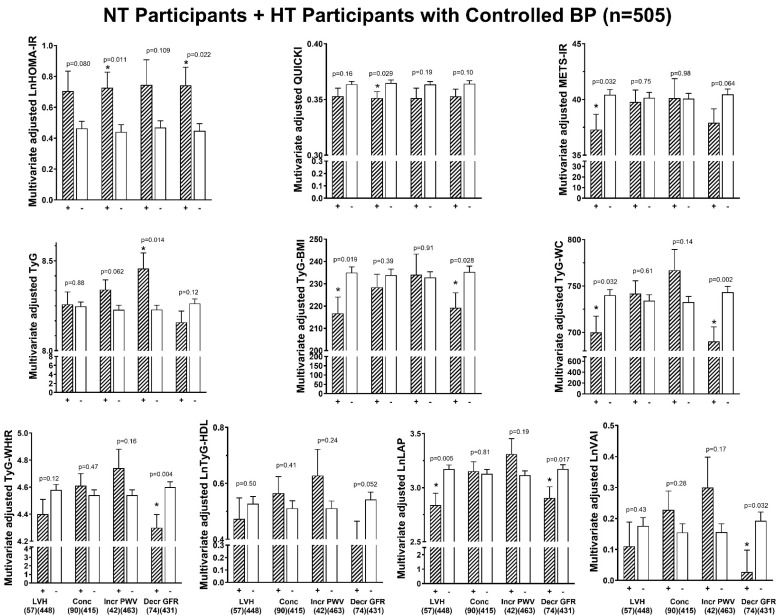
Adjusted mean values (±SEM) of various indices of insulin resistance in those with (+) compared to those without (-) end-organ damage (adjustments are for age, sex, regular alcohol intake, regular tobacco intake, treatment for hypertension, heart rate, and brachial pulse pressure) in the normotensive (NT) participants plus the hypertensive (HT) participants with controlled blood pressure (BP) (n = 505). BMI, body mass index; Conc, increased left ventricular relative wall thickness; Decr, decreased; GFR, estimated glomerular filtration rate; HDL, high-density lipoprotein; Incr, increased; lnHOMA-IR, natural logarithm of homeostatic model assessment for insulin resistance; lnLAP, natural logarithm of lipid accumulation product; LDL, low-density lipoprotein; LVH, increased left ventricular mass indexed to body surface area; METS-IR, metabolic score for insulin resistance; PWV, pulse wave velocity; QUICKI, quantitative insulin sensitivity check index; TyG, triglyceride–glucose index; TyG-BMI, triglyceride–body mass index; lnTyG-HDL, natural logarithm of triglycerides to high-density cholesterol concentrations; TyG-WC, triglyceride–waist circumference; TyG-WHtR, triglyceride–waist-to-height ratio; lnVAI, natural logarithm of visceral adiposity index. * *p* < 0.05.

Figure 4 (all the participants), Figure 5 (the participants without DM) and Figure 6 (the normotensive participants plus the hypertensive participants with controlled BP) show adjusted mean values of the indices of IR in the participants with compared to those without end-organ damage. After adjustments for confounders in all the participants, only HOMA-IR was consistently higher and QUICKI consistently lower in those participants with compared to those participants without increased LVMI, increased LV RWT (concentricity), and decreased GFR (Figure 4). In all the participants, HOMA-IR tended to be higher in those with increased PWV (*p* = 0.102, Figure 4). No other indices of IR were higher in those with compared to those without increased LVMI, increased LV RWT, decreased GFR, or increased PWV (Figure 4). TyG-WC and TyG-WHtR were lower in the participants with compared to without decreased GFR. Similarly, in the participants without DM (Figure 5), HOMA-IR adjusted for confounders was higher and QUICKI was lower in those participants with compared to those without increased LVMI, increased LV RWT (concentricity), and decreased GFR. In addition, in the participants without DM, no other indices of IR were higher in those with compared to those without increased LVMI, increased LV RWT, decreased GFR, or increased PWV (Figure 5). LAP was lower in those with compared to those without increased LVMI (Figure 5). In the normotensive participants plus the hypertensive participants with controlled BP (Figure 6), HOMA-IR adjusted for confounders was numerically higher in those participants with compared to those without increased LVMI (trend, *p* = 0.08), increased LV RWT (concentricity), and decreased GFR (Figure 6). QUICKI adjusted for confounders was only lower in those with increased LV RWT (concentricity) (Figure 6). TyG adjusted for confounders was only increased in those with increased PWV (Figure 6).

### 3.7. HOMA-IR and Odds of End-Organ Damage Independent of CVS Risk Factors

One standard deviation increase in HOMA-IR or decrease in QUICKI was associated with greater odds of increased LVMI, increased LV RWT, increased PWV (trend for HOMA-IR, *p* = 0.0557), and decreased GFR (Table 4). Similarly, in the cohort without DM, a unit increase in HOMA-IR was associated with greater odds of increased LVMI, increased LV RWT, and decreased GFR (Table 4). Similar data were obtained in the normotensive participants plus the hypertensive participants with controlled BP (Table 4). After including additional CVS risk factors in the models, one standard deviation increase in HOMA-IR or decrease in QUICKI remained associated with greater odds of increased LVMI, increased LV RWT, and decreased GFR but not with increased PWV (Table 4). Similarly, in the cohort without DM, and in the normotensive participants plus the hypertensive participants with controlled BP, one standard deviation increase in HOMA-IR or decrease in QUICKI was associated with greater odds of increased LVMI, increased LV RWT, and decreased GFR, independent of additional CVS risk factors (Table 4). Importantly, in all the participants (Table 5), in those without DM (Table S9), and in the normotensive participants plus the hypertensive participants with controlled BP (Table S10), HOMA-IR and QUICKI were second only to age in association with increased LV RWT and decreased GFR, third only to age and BP in association with increased LVMI, and fourth to age, BP, and HR in association with increased PWV. Notably, the measures of adiposity (BMI and waist circumference) were not positively significantly associated with end-organ damage in the models (Table 5, Tables S9 and S10). Furthermore, triglycerides were not positively, and HDL cholesterol was not negatively, significantly associated with end-organ damage in the models (Table 5, Tables S9 and S10).

## 4. Discussion

In a community study of predominantly young participants of African ancestry, we showed that HOMA-IR and QUICKI were the only indexes of IR that were consistently associated with most of the assessed end-organ measures (LV mass index, LV relative wall thickness, E/A, E/e’, e’, PWV, GFR), independent of confounders. Furthermore, only HOMA-IR and QUICKI were higher in those with compared to those without end-organ damage (LV hypertrophy, concentric LV, reduced GFR), independent of confounders. Importantly, the associations between either HOMA-IR or QUICKI and the end-organ measures were independent of additional CVS risk factors, including adiposity indices, and replicated in the participants without diabetes mellitus, in the normotensive participants plus the hypertensive participants with controlled BP, and in the men and women separately.

Our data showing that HOMA-IR and QUICKI are the best indexes of IR to use when assessing cardiovascular risk in people of African ancestry differ from those reported in other populations. In a predominantly (70.8%) Non-Hispanic White race group (NHANES 2001–2018), METS-IR, but neither the TyG index, the TyG-HDL ratio, nor HOMA-IR, was associated with all-cause and CVD mortality in the general population (14% with DM), and particularly in young adults (aged < 65 years) [12]. In contrast, in young (aged 35–64 years) Hispanic and non-Hispanic White individuals without DM, the TyG index was preferable to HOMA-IR in predicting the risk of CHD [13]. Moreover, the strength of the relationship between these indices of IR and the risk of CHD was found to differ across Hispanic and non-Hispanic populations [13]. In Chinese middle-aged and older populations, the Chinese VAI was superior to other indices (TyG index, LAP, TyG-BMI, TyG-WC) in predicting cardiometabolic multimorbidity (co-occurrence of stroke, DM, and/or hyperglycaemia) [29]. Our data, therefore, support the concept that the efficacy of IR indices in predicting cardiovascular risk is likely to differ across populations and race groups [13,18].

It is not surprising that our data differ from those of NHANES 2001–2018, as in NHANES, only 10.5% were of the Non-Hispanic Black race group [12]. Indeed, our data support those reported in Black Africans from the Democratic Republic of Congo, where only IR and obesity were independent determinants of left ventricular hypertrophy [30]. Importantly, in this case–control study, the association between HOMA-IR and the left ventricular mass index was independent of age, duration of hypertension, BMI, waist circumference, and plasma glucose and insulin concentrations [30]. However, only HOMA-IR and no other indices of IR were assessed in this study [30]. Furthermore, LVM was the only end-organ measure assessed [30]. Hence, to our knowledge, our data are the first to compare, in Black Africans, the associations between a large number of indices of IR and a diverse group of end-organ measures.

There are a number of potential explanations for the disparity in the efficacy of IR indices in predicting cardiovascular risk in Black Africans compared to other race groups. There are differences in lipid profiles between Non-Hispanic White and Black individuals [18]; hence, the predictive efficacy of indices of IR, which rely on triglyceride and HDL-cholesterol concentrations, are likely to differ between these race groups. Similarly, in South Africa, individuals of African ancestry are reported to have lower plasma concentrations of total and LDL cholesterol and triglycerides compared to White individuals [31]. Moreover, in South Africa, Black individuals, compared to their White counterparts, have higher HDL cholesterol and lower triglyceride concentrations, despite greater insulin resistance [32]. It has, therefore, been suggested that indices of IR, especially those that rely on lipid levels, may underestimate cardiovascular risk in Black individuals [18]. Indeed, a recent systematic review reported that the TyG-HDL ratio is a poor surrogate of IR in African Americans, probably due to their low triglyceride levels [33]. In keeping with these findings, in our study, we show that IR indices that rely on triglycerides (METS-IR, TyG index, TyG-BMI, TyG-WC, TyG-WHtR, LAP, VAI) and HDL cholesterol (METS-IR, TyG-HDL, VAI) were not associated with any of the end-organ measures other than E/A.

In addition to differences in lipid profiles, individuals of African ancestry from Sub-Saharan Africa, compared to Europeans, have low levels of visceral and high levels of abdominal and gluteo-femoral subcutaneous adipose tissue despite a higher prevalence of obesity [34]. It is possible that these differences in adipose tissue distribution may impact racial differences in the efficacy of IR indices relying on BMI and waist circumference. In the current study, we show that IR indices that include either BMI (METS-IR, TyG-BMI, VAI) or waist circumference (TyG-WC, TyG-WHtR, LAP, VAI) were not associated with any of the end-organ measures other than E/A.

The reasons for the independent relationships between E/A and all the indices of IR in the current study are unclear. However, in the current study, the relationships between the E/A and IR indices were strongest for those indices of IR that included either BMI or waist circumference (METS-IR, TyG-BMI, TyG-WC, TyG-WHtR, LAP). In this regard, previous studies have reported a strong inverse association between BMI and LV diastolic function independent of LV mass and associated risk factors (age, sex, heart rate, hypertension, and DM) [35].

Africans from Sub-Saharan Africa have a higher prevalence of obesity, insulin resistance, and hyperinsulinaemia compared to Europeans [34]. This hyperinsulinaemia that is characteristic of Africans is driven primarily by increased insulin secretion and decreased hepatic insulin clearance, which ultimately lead to beta cell exhaustion [34]. Moreover, this hyperinsulinaemia in Africans is independent of adiposity and insulin sensitivity and is a highly conserved trait, being reported in indigenous and diasporic Black African adults and children [34]. The presence of this hyperinsulinaemic trait in Africans would explain our findings that HOMA-IR and QUICKI are the only indexes of IR that are consistently associated with cardiovascular risk in Africans. Strikingly, in this regard, and in line with these previously reported findings, 69.2% of the participants in the current study had impaired glucose metabolism (14.1% had DM, and 55.1% of the participants without DM had pre-DM). It is important to note that in assessing cardiovascular risk, we showed that HOMA-IR and QUICKI are of greater value than using either insulin or blood glucose alone.

The mechanisms by which insulin resistance contributes to cardiovascular risk are inflammation, endothelial dysfunction, and oxidative stress [36,37]. Although hyperinsulinaemia itself could contribute to end-organ damage, we showed that end-organ damage is not only attributed to hyperinsulinaemia but also to increases in blood glucose. Some end organs seem to be more susceptible to blood glucose levels (e.g., PWV), some are affected more by insulin (e.g., LVMI, E/A, GFR), whereas others are affected by both insulin and blood glucose (e.g., LV RWT, E/e’, e’). Hence, in Black individuals, it seems that insulin and insulin resistance, as well as blood glucose, can cause end-organ changes.

As the current data were collected in an urban population of individuals of African ancestry, they may not necessarily be applicable to all populations of African ancestry. In this regard, the characteristics of people with DM differ between those living in urban versus rural settings [34]. Notably, those living in rural settings have lower socioeconomic status and younger age of onset of DM [34]. Additional factors that may influence the relationships between the indices of IR and end-organ measures are socioeconomic status, dietary habits, and genetic predisposition, which have been shown to influence the pathogenesis of DM in Sub-Saharan Africans [34]. In this regard, socioeconomic status is positively associated with DM prevalence in Sub-Saharan Africa [38], and a high reliance on processed carbohydrate-rich foods in combination with a genetic predisposition may drive hypersecretion of insulin and decreased insulin clearance [34].

The present study has several limitations. The study design was cross-sectional; hence, the relationships noted may not represent cause and effect. Longitudinal studies evaluating the predictive ability of IR indices for cardiovascular risk and mortality in people of African ancestry are therefore required. However, the present study provides critical evidence in support of such future studies. Second, the proportions of individuals with end-organ damage were small (<35%). Nevertheless, we were consistently able to show significantly increased levels of HOMA-IR in those with compared to those without end-organ damage. Third, as two-thirds of our participants were women, the results may pertain more to women than to men. However, we were able to replicate the main findings in sex-specific analyses. Fourth, as no data on socioeconomic status, dietary habits, or genetic predisposition were available in the current study, we were unable to assess the impact of these factors on the relationships observed. This issue should be addressed in future studies. The strengths of our study include the replication of our findings in a cohort without DM, in normotensive participants plus hypertensive participants with controlled BP, and the comparison of ten recommended indices of IR.

In conclusion, in the present study, in predominantly young individuals of African ancestry, we showed that of ten recommended indices of IR, only HOMA-IR and QUICKI were consistently associated with end-organ measures, independent of confounders and cardiovascular risk factors. Furthermore, we showed that only HOMA-IR was increased and QUICKI was decreased in individuals with compared to individuals without end-organ damage. These data were replicated in participants without DM and in normotensive participants plus hypertensive participants with controlled BP, as well as separately in men and women. As both insulin and blood glucose were separately associated with different end-organ measures, HOMA-IR and QUICKI are of greater value than using either insulin or blood glucose alone in assessing cardiovascular risk in individuals of African ancestry. Future prospective studies assessing whether HOMA-IR and QUICKI translate into increased cardiovascular event rates in individuals of African ancestry are required.

## Figures and Tables

**Table 1:** Characteristics of the study participants.

	All	Without DM
Sample size	779	669
% Female (n)	65.1 (508)	64.3 (430)
Age (years)	45.0 ± 18.3	42.3 ± 17.8
% <65 years of age (n)	82.8 (645)	86.1 (576)
Body mass index (kg/m^2^)	29.0 ± 7.4	28.3 ± 7.3
Waist circumference (cm)	91.7 ± 16.7	89.6 ± 16.2
% Overweight (n)	25.3 (197)	25.3 (169)
% Obese (n)	41.3 (322)	37.2 (249)
Brachial SBP (mm Hg)	128 ± 22	126 ± 22
Brachial DBP (mm Hg)	83 ± 12	82 ± 12
% Hypertension (n)	46.7 (364)	40.1 (268)
% Uncontrolled BP (n)	35.2 (274)	32.6 (218)
% HT with uncontrolled BP (n)	75.3 (274/364)	81.3 (218/268)
% Treated for hypertension (n)	26.6 (207)	19.6 (131)
% HT treated for HT (n)	56.9 (207/364)	48.9 (131/268)
Heart rate (beats/min)	65.9 ± 11.5	65.1 ± 11.1
% Regular tobacco intake (n)	15.4 (120)	16.4 (110)
% Regular alcohol intake (n)	21.2 (165)	22.7 (152)
% Diabetes mellitus (n)	14.2 (110)	0
**Fasting plasma concentrations**		
Glucose (mmol/L)	4.60 (4.20 to 5.30)	4.50 (4.20 to 5.00) ***
Glycated haemoglobin (%)	5.80 (5.50 to 6.10)	5.70 (5.50 to 5.96) ***
Insulin (μU/mL)	7.66 (4.02 to 14.00)	7.03 (3.77 to 13.30)
Total cholesterol (mmol/L)	4.60 (4.00 to 5.21)	4.50 (4.00 to 5.16)
LDL cholesterol (mmol/L)	2.60 (2.20 to 3.19)	2.57 (2.15 to 3.18)
HDL cholesterol (mmol/L)	1.40 (1.20 to 1.50)	1.40 (1.24 to 1.60)
Triglycerides (mmol/L)	1.07 (0.80 to 1.51)	1.00 (0.70 to 1.47)
**Insulin resistance indices**		
HOMA-IR	1.61 (0.83 to 3.42)	1.43 (0.76 to 2.80)
QUICKI	0.36 ± 0.06	0.37 ± 0.06 *
METS-IR	41.9 ± 12.3	40.1 ± 11.6 **
TyG	8.36 ± 0.67	8.23 ± 0.55 ***
TyG-BMI	244.2 ± 69.5	234.3 ± 66.0 *
TyG-WC	771.1 ± 174.4	741.1 ± 161.2 **
TyG-WHtR	4.79 ± 1.14	4.60 ± 1.06 **
TyG-HDL	1.72 (1.21 to 2.67)	1.57 (1.14 to 2.56)
LAP	32.2 (13.5 to 60.9)	27.2 (12.0 to 52.5) *
VAI	1.27 (0.80 to 2.03)	1.16 (0.76 to 1.83)
**End-organ measures**		
LVMI (g/m^2^)	75.3 ± 29.6	74.3 ± 29.1
% LV hypertrophy (n)	16.1 (125)	15.0 (100)
LV relative wall thickness	0.38 ± 0.08	0.37 ± 0.07
% Concentricity of LV	22.1 (172)	20.3 (136)
LV midwall fractional shortening	0.26 ± 0.08	0.26 ± 0.08
EF (%)	67.2 ± 8.8	67.2 ± 8.6
E/A	1.29 ± 0.50	1.33 ± 0.49
E/e’ (n)	7.38 ± 4.04 (516)	7.03 ± 3.68 (414)
e’ (n)	11.48 ± 4.04 (516)	12.02 ± 3.98 (414) *
GFR (ml/min per 1.73 m^2^)	98.13 ± 21.29	100.47 ± 20.36 *
% Decreased GFR (n)	35.2 (274)	32.7 (219)
Pulse wave velocity (m/s)	6.09 ± 2.65	5.77 ± 2.36 *
% Increased PWV (n)	17.1 (133)	12.7 (85)

Data are shown as mean ± SD, median (interquartile range), or percentages. BP, blood pressure; DBP, diastolic blood pressure; E/A, early-to-late mitral velocity; e’, myocardial tissue lengthening in early diastole at the mitral annulus; E/e’, transmitral early blood flow velocity/velocity of the mean value of lateral and septal wall myocardial tissue lengthening in early diastole at the mitral annulus; EF, ejection fraction; GFR, estimated glomerular filtration rate; HDL, high-density lipoprotein; HOMA-IR, homeostatic model assessment for insulin resistance; HT, hypertension; LAP, lipid accumulation product; LDL, low-density lipoprotein; LV, left ventricle; LVMI, left ventricular mass indexed to body surface area; METS-IR, metabolic score for insulin resistance; PWV, pulse wave velocity; QUICKI, quantitative insulin sensitivity check index; SBP, systolic blood pressure; TyG, triglyceride–glucose index; TyG-BMI, triglyceride–body mass index; TyG-HDL, triglycerides-to-high-density cholesterol concentrations; TyG-WC, triglyceride–waist circumference; TyG-WHtR, triglyceride–waist-to-height ratio; VAI, visceral adiposity index. * *p* < 0.05, ** *p* < 0.005, *** *p* < 0.0001 versus all the participants.

**Table 2:** Heat map showing unadjusted correlations (Pearson’s r values) between various indices of insulin resistance (n = 779).

	lnHOMA-IR	QUICKI	METS-IR	TyG	TyG-BMI	TyG-WC	TyG-WHtR	lnTyG-HDL	lnLAP	
QUICKI	−0.982									Pearson’s Correlation
METS-IR	0.334	−0.340								
TyG	0.402	−0.375	0.498							0.501 to 1.000
TyG-BMI	0.313	−0.321	0.965	0.510						0.001 to 0.500
TyG-WC	0.349	−0.348	0.847	0.690	0.874					0
TyG-WHtR	0.331	−0.335	0.860	0.651	0.894	0.974				−0.001 to −0.500
lnTyG-HDL	0.283	−0.269	0.512	0.856	0.437	0.612	0.565			−0.501 to −1.000
lnLAP	0.304	−0.306	0.769	0.717	0.804	0.912	0.894	0.683		
lnVAI	0.304	-0.297	0.600	0.839	0.541	0.715	0.695	0.955	0.789	*p* < 0.0001 for all

All correlations are significant at *p* < 0.0001. lnHOMA-IR, natural logarithm of homeostatic model assessment for insulin resistance; lnLAP, natural logarithm of lipid accumulation product; METS-IR, metabolic score for insulin resistance; QUICKI, quantitative insulin sensitivity check index; TyG, triglyceride–glucose index; TyG-BMI, triglyceride–body mass index; lnTyG-HDL, natural logarithm of triglycerides to high-density cholesterol concentrations; TyG-WC, triglyceride–waist circumference; TyG-WHtR, triglyceride–waist-to-height ratio; lnVAI, natural logarithm of visceral adiposity index.

**Table 3:** Unadjusted relationships between various indices of insulin resistance and end-organ measures in all the participants (n = 779).

	r (95%CI)	*p*-Value	r (95%CI)	*p*-Value	r (95%CI)	*p*-Value
	**LVMI**		**LV RWT**		**EF**	
lnHOMA-IR	0.140 (0.070 to 0.208)	**<0.0001**	0.204 (0.136 to 0.270)	**<0.0001**	0.002 (−0.068 to 0.072)	=0.9553
QUICKI	−0.110 (−0.179 to −0.040)	**=0.0020**	−0.182 (−0.249 to −0.114)	**<0.0001**	0.005 (−0.066 to 0.075)	=0.8984
METS-IR	0.076 (0.005 to 0.145)	**=0.0347**	0.176 (0.107 to 0.243)	**<0.0001**	−0.008 (−0.078 to 0.063)	=0.8327
TyG	0.125 (0.055 to 0.193)	**=0.0005**	0.175 (0.106 to 0.242)	**<0.0001**	0.023 (−0.047 to 0.093)	=0.5221
TyG-BMI	0.088 (0.018 to 0.158)	**=0.0136**	0.179 (0.110 to 0.246)	**<0.0001**	−0.002 (−0.072 to 0.068)	=0.9567
TyG-WC	0.145 (0.076 to 0.213)	**<0.0001**	0.185 (0.116 to 0.252)	**<0.0001**	−0.002 (−0.072 to 0.068)	=0.9577
TyG-WHtR	0.142 (0.072 to 0.210)	**<0.0001**	0.212 (0.144 to 0.278)	**<0.0001**	0.013 (−0.057 to 0.084)	=0.7098
lnTyG-HDL	0.087 (0.017 to 0.156)	**=0.0150**	0.110 (0.040 to 0.179)	**=0.0021**	0.025 (−0.046 to 0.095)	=0.4890
lnLAP	0.104 (0.033 to 0.173)	**=0.0039**	0.167 (0.098 to 0.235)	**<0.0001**	0.009 (−0.062 to 0.079)	=0.8120
lnVAI	0.058 (−0.012 to 0.128)	=0.1046	0.131 (0.061 to 0.199)	**=0.0002**	0.034 (−0.037 to 0.104)	=0.3457
	**mFS**		**E/A**		**E/e’ (n = 515)**	
lnHOMA-IR	−0.054 (−0.124 to 0.016)	=0.1292	−0.198 (−0.264 to −0.129)	**<0.0001**	0.182 (0.097 to 0.264)	**<0.0001**
QUICKI	0.035 (−0.035 to 0.105)	=0.3282	0.181 (0.112 to 0.248)	**<0.0001**	−0.184 (−0.266 to −0.099)	**<0.0001**
METS-IR	−0.013 (−0.083 to 0.058)	=0.7256	−0.389 (−0.447 to −0.327)	**<0.0001**	0.290 (0.208 to 0.366)	**<0.0001**
TyG	−0.045 (−0.115 to 0.025)	=0.2067	−0.384 (−0.442 to −0.322)	**<0.0001**	0.175 (0.090 to 0.257)	**<0.0001**
TyG-BMI	−0.036 (−0.106 to 0.035)	=0.3194	−0.417 (−0.473 to −0.357)	**<0.0001**	0.308 (0.227 to 0.384)	**<0.0001**
TyG-WC	−0.052 (−0.122 to 0.018)	=0.1476	−0.483 (−0.534 to −0.427)	**<0.0001**	0.296 (0.215 to 0.372)	**<0.0001**
TyG-WHtR	−0.054 (−0.123 to 0.017)	=0.1357	−0.501 (−0.552 to −0.446)	**<0.0001**	0.337 (0.258 to 0.411)	**<0.0001**
lnTyG-HDL	0.021 (−0.049 to 0.091)	=0.5601	−0.301 (−0.363 to −0.236)	**<0.0001**	0.111 (0.025 to 0.195)	**=0.0115**
lnLAP	−0.061 (−0.131 to 0.009)	=0.0891	−0.470 (−0.523 to −0.413)	**<0.0001**	0.262 (0.179 to 0.341)	**<0.0001**
lnVAI	0.013 (−0.057 to 0.083)	=0.7192	−0.355 (−0.415 to −0.292)	**<0.0001**	0.171 (0.086 to 0.253)	**<0.0001**
	**e’ (n = 515)**		**PWV**		**GFR**	
lnHOMA-IR	−0.219 (−0.299 to −0.135)	**<0.0001**	0.149 (0.080 to 0.217)	**<0.0001**	−0.253 (−0.318 to −0.186)	**<0.0001**
QUICKI	0.205 (0.121 to 0.286)	**<0.0001**	−0.127 (−0.196 to −0.057)	**= 0.0004**	0.235 (0.167 to 0.300)	**< 0.0001**
METS-IR	−0.449 (−0.515 to −0.377)	**<0.0001**	0.250 (0.183 to 0.314)	**<0.0001**	−0.322 (−0.384 to −0.258)	**<0.0001**
TyG	−0.430 (−0.497 to −0.357)	**<0.0001**	0.353 (0.290 to 0.413)	**<0.0001**	−0.346 (−0.406 to −0.282)	**<0.0001**
TyG-BMI	−0.475 (−0.539 to −0.405)	**<0.0001**	0.279 (0.213 to 0.343)	**<0.0001**	−0.347 (−0.407 to −0.283)	**<0.0001**
TyG-WC	−0.533 (−0.591 to −0.467)	**<0.0001**	0.379 (0.317 to 0.437)	**<0.0001**	−0.404 (−0.461 to −0.344)	**<0.0001**
TyG-WHtR	−0.555 (−0.612 to −0.492)	**<0.0001**	0.385 (0.323 to 0.443)	**<0.0001**	−0.412 (−0.468 to −0.352)	**<0.0001**
lnTyG-HDL	−0.355 (−0.428 to −0.277)	**<0.0001**	0.240 (0.173 to 0.305)	**<0.0001**	−0.256 (−0.320 to −0.189)	**<0.0001**
lnLAP	−0.499 (−0.562 to −0.430)	**<0.0001**	0.368 (0.305 to 0.427)	**<0.0001**	−0.417 (−0.474 to −0.357)	**<0.0001**
lnVAI	−0.407 (−0.476 to −0.332)	**<0.0001**	0.266 (0.199 to 0.330)	**<0.0001**	−0.299 (−0.362 to −0.234)	**<0.0001**

All significant associations are shown in bold type. CI, confidence interval; E/A, early-to-late mitral velocity; e’, myocardial tissue lengthening in early diastole at the mitral annulus; E/e’, transmitral early blood flow velocity/velocity of the mean value of lateral and septal wall myocardial tissue lengthening in early diastole at the mitral annulus; EF, ejection fraction; GFR, estimated glomerular filtration rate; lnHOMA-IR, natural logarithm of homeostatic model assessment for insulin resistance; lnLAP, natural logarithm of lipid accumulation product; LVMI, left ventricular mass indexed to body surface area; LV RWT, left ventricular relative wall thickness; METS-IR, metabolic score for insulin resistance; mFS, left ventricular midwall fractional shortening; PWV, pulse wave velocity; QUICKI, quantitative insulin sensitivity check index; r, Pearson’s correlation coefficient; TyG, triglyceride–glucose index; TyG-BMI, triglyceride–body mass index; lnTyG-HDL, natural logarithm of triglycerides to high-density cholesterol concentrations; TyG-WC, triglyceride–waist circumference; TyG-WHtR, triglyceride–waist-to-height ratio; lnVAI, natural logarithm of visceral adiposity index.

**Table 4:** Odds of end-organ damage in association with one SD increment in HOMA-IR or QUICKI in all the participants (n = 779), in the participants without DM (n = 669), and in the NT participants and the HT participants with controlled BP (n = 505).

	OR (95%CI)	*p*-Value	OR (95%CI)	*p*-Value	OR (95%CI)	*p*-Value
	Model 1		Model 2		Model 3	
HOMA-IR vs.	All Participants (n = 779)					
LVH-BSA	1.219 (1.029–1.439)	**=0.0192**	1.261 (1.055–1.506)	**=0.0099**	1.260 (1.055–1.506)	**=0.0100**
Concentricity	1.189 (1.018–1.392)	**=0.0281**	1.199 (1.020–1.411)	**=0.0272**	1.199 (1.020–1.412)	**=0.0268**
Incr PWV	1.197 (0.994–1.442)	=0.0557	1.147 (0.947–1.391)	=0.1574	1.153 (0.951–1.397)	=0.1426
Decr GFR	1.272 (1.075–1.513)	**=0.0054**	1.374 (1.148–1.655)	**=0.0006**	1.367 (1.141–1.647)	**=0.0008**
QUICKI vs.						
LVH-BSA	0.772 (0.622–0.952)	**=0.0169**	0.737 (0.587–0.919)	**=0.0073**	0.737 (0.587–0.919)	**=0.0073**
Concentricity	0.813 (0.676–0.974)	**=0.0262**	0.802 (0.661–0.970)	**=0.0240**	0.800 (0.660–0.967)	**=0.0224**
Incr PWV	0.829 (0.655–1.045)	=0.1149	0.874 (0.680–1.120)	=0.2872	0.865 (0.674–1.108)	=0.2526
Decr GFR	0.739 (0.597–0.909)	**=0.0047**	0.660 (0.525–0.824)	**=0.0003**	0.668 (0.532–0.834)	**=0.0004**
HOMA-IR vs.	Participants without DM (n = 669)					
LVH-BSA	1.306 (1.092–1.564)	**=0.0032**	1.343 (1.114–1.627)	**=0.0019**	1.342 (1.113–1.625)	**=0.0019**
Concentricity	1.205 (1.017–1.426)	**=0.0284**	1.225 (1.026–1.463)	**=0.0230**	1.227 (1.028–1.464)	**=0.0217**
Incr PWV	1.110 (0.873–1.364)	=0.3468	1.040 (0.802–1.299)	=0.7448	1.048 (0.809–1.309)	=0.6929
Decr GFR	1.290 (1.076–1.552)	**=0.0060**	1.407 (1.160–1.718)	**=0.0006**	1.398 (1.151–1.709)	**=0.0008**
QUICKI vs.						
LVH-BSA	0.683 (0.537–0.863)	**=0.0017**	0.657 (0.510–0.839)	**=0.0009**	0.654 (0.508–0.835)	**=0.0008**
Concentricity	0.823 (0.674–1.003)	=0.0549	0.814 (0.660–0.999)	=0.0508	0.810 (0.657–0.995)	**=0.0464**
Incr PWV	0.899 (0.686–1.177)	=0.4376	0.976 (0.729–1.308)	=0.8712	0.965 (0.721–1.293)	=0.8112
Decr GFR	0.744 (0.587–0.937)	**=0.0130**	0.663 (0.514–0.849)	**=0.0013**	0.669 (0.519–0.857)	**=0.0017**
HOMA-IR vs.	NT Participants + HT Participants with Controlled BP (n = 505)					
LVH-BSA	1.205 (0.959–1.490)	=0.0892	1.215 (0.955–1.520)	=0.0934	1.204 (0.949–1.504)	=0.1059
Concentricity	1.267 (1.039–1.547)	**=0.0176**	1.252 (1.021–1.535)	**=0.0278**	1.249 (1.020–1.531)	**=0.0294**
Incr PWV	1.195 (0.898–1.546)	=0.1865	1.119 (0.823–1.468)	=0.4343	1.124 (0.827–1.473)	=0.4167
Decr GFR	1.426 (1.152–1.779)	**=0.0011**	1.626 (1.295–2.072)	**<0.0001**	1.619 (1.288–2.065)	**<0.0001**
QUICKI vs.						
LVH-BSA	0.796 (0.590–1.065)	=0.1258	0.775 (0.562–1.058)	=0.1126	0.774 (0.563–1.055)	=0.1095
Concentricity	0.763 (0.596–0.971)	**=0.0298**	0.771 (0.597–0.989)	**=0.0431**	0.756 (0.585–0.971)	**=0.0302**
Incr PWV	0.777 (0.518–1.149)	=0.2114	0.905 (0.590–1.377)	=0.6443	0.881 (0.575–1.337)	=0.5538
Decr GFR	0.776 (0.580–1.031)	=0.0844	0.654 (0.473–0.891)	**=0.0083**	0.667 (0.484–0.908)	**=0.0114**

One SD for HOMA-IR: 4.39 (n = 779), 4.07 (n = 669), 3.80 (n = 505). One SD for QUICKI: 0.055 (n = 779), 0.055 (n = 669), 0.054 (n = 505). All significant associations are shown in bold type. Model 1: Adjustments for age, sex, regular alcohol intake, regular tobacco intake, treatment for hypertension, heart rate, and brachial pulse pressure. Model 2: Adjustments as for Model 1 plus adjustments for triglycerides, HDL cholesterol, brachial SBP and DBP (instead of PP), and waist circumference. Model 3: Adjustments as for Model 1 plus adjustments for triglycerides, HDL cholesterol, brachial SBP and DBP (instead of PP), and BMI. CI, confidence interval; Concentricity, increased left ventricular relative wall thickness; Decr, decreased; GFR, estimated glomerular filtration rate; Incr, increased; HOMA-IR, homeostatic model assessment for insulin resistance; HT, hypertensive; LVH-BSA, increased left ventricular mass indexed to body surface area; NT, normotensive; OR, odds ratio; PWV, pulse wave velocity; QUICKI, quantitative insulin sensitivity check index.

**Table 5:** Wald Χ^2^ for HOMA-IR or QUICKI compared to measures of adiposity (BMI and waist circumference), triglycerides, HDL cholesterol, and all other significant determinants of end-organ damage in all the participants (n = 779).

	Wald Χ^2^	*p*-Value	Wald Χ^2^	*p*-Value	Wald Χ^2^	*p*-Value
	Model 1		Model 2		Model 3	
LVH-BSA vs.						
HOMA-IR	5.48	**=0.0192**	6.65	**=0.0099**	6.63	**=0.0100**
Age	12.72	**=0.0004**	13.17	**=0.0003**	13.62	**=0.0002**
BP *	13.31	**=0.0003**	11.65	**=0.0006**	11.70	**=0.0006**
HR	6.05	**=0.0139**	5.86	**=0.0155**	5.93	**=0.0148**
WC	—	—	−0.13	=0.7225	—	—
BMI	—	—	—	—	−0.12	=0.7242
Triglycerides	—	—	−3.45	=0.0631	−3.62	=0.0571
HDL cholesterol	—	—	−0.06	=0.8104	−0.05	=0.8171
LVH-BSA vs.						
QUICKI	−5.71	**=0.0169**	−7.19	**=0.0073**	−7.20	**=0.0073**
Age	13.06	**=0.0003**	13.82	**=0.0002**	14.17	**=0.0002**
BP *	14.22	**=0.0002**	12.63	**=0.0004**	12.71	**=0.0004**
HR	−6.26	**=0.0123**	−6.00	**=0.0143**	−6.12	**=0.0134**
WC	—	—	−0.30	=0.5852	—	—
BMI	—	—	—	—	−0.32	=0.5720
Triglycerides	—	—	−3.38	=0.0660	−3.61	=0.0573
HDL cholesterol	—	—	−0.01	=0.9088	−0.01	=0.9166
Concentricity vs.						
HOMA-IR	4.82	**=0.0281**	4.88	**=0.0272**	4.90	**=0.0268**
Age	11.51	**=0.0007**	10.96	**=0.0009**	11.77	**=0.0006**
WC	—		−0.13	=0.7225	—	—
BMI	—	—	—	—	−0.34	=0.5621
Triglycerides		−0.96	=0.3265	−1.00	=0.3168	−0.96
HDL cholesterol	—	—	−0.22	=0.6357	−0.26	=0.6109
Concentricity vs.						
QUICKI	−4.94	**=0.0262**	−5.09	**=0.0240**	−5.21	**=0.0224**
Age	11.88	**=0.0006**	11.58	**=0.0007**	12.31	**=0.0004**
WC		—	−0.27	=0.6013	—	—
BMI	—	—	—	—	−0.58	=0.4449
Triglycerides		−0.88	=0.3467	−0.94	=0.3316	
HDL cholesterol	—	—	−0.11	=0.7376	−0.13	=0.7151
Incr PWV vs.						
HOMA-IR	3.66	=0.0557	2.00	=0.1574	2.15	=0.1426
Age	65.59	**<0.0001**	60.85	**<0.0001**	63.87	**<0.0001**
BP *	20.11	**<0.0001**	15.78	**<0.0001**	15.88	**<0.0001**
HR	8.14	**=0.0043**	6.45	**=0.0111**	6.63	**=0.0100**
WC	—	—	0.32	=0.5716	—	—
BMI	—	—	—	—	0.01	=0.9165
Triglycerides	—	—	0.17	=0.6793	0.20	=0.6563
HDL cholesterol	—	—	−0.16	=0.6918	−0.25	=0.6172
Incr PWV vs.						
QUICKI	−2.48	=0.1149	−1.13	=0.2872	−1.31	=0.2526
Age	66.19	**<0.0001**	61.09	**<0.0001**	64.11	**<0.0001**
BP *	20.17	**<0.0001**	15.47	**<0.0001**	15.59	**<0.0001**
HR	7.83	**=0.0051**	6.32	**=0.0119**	6.46	**=0.0110**
WC	—	—	0.27	=0.5999	—	—
BMI	—	—	—	—	0.002	=0.9658
Triglycerides	—	—	0.21	=0.6449	0.24	=0.6264
HDL cholesterol	—	—	−0.14	=0.7062	−0.22	=0.6368
Decr GFR vs.						
HOMA-IR	7.74	**=0.0054**	11.75	**=0.0006**	11.23	**=0.0008**
Age	41.26	**<0.0001**	52.07	**<0.0001**	49.59	**<0.0001**
WC	—	—	−4.56	**=0.0327**	—	—
BMI	—	—	—	—	−1.77	=0.1839
Triglycerides	—	—	−3.48	=0.0621	−4.32	**=0.0377**
HDL cholesterol	—	—	−0.05	=0.8311	0.001	=0.9959
Decr GFR vs.						
QUICKI	−8.00	**=0.0047**	−13.08	**=0.0003**	−12.40	**=0.0004**
Age	41.74	**<0.0001**	53.11	**<0.0001**	50.31	**<0.0001**
WC	—	—	−5.69	**=0.0171**	—	—
BMI	—	—	—	—	−2.56	=0.1096
Triglycerides	—	—	−3.23	=0.0724	−4.13	**=0.0420**
HDL cholesterol	—	—	0.001	=0.9807	0.049	=0.8242

All significant associations are shown in bold type. Model 1: Adjustments for age, sex, regular alcohol intake, regular tobacco intake, treatment for hypertension, heart rate, and brachial pulse pressure. Model 2: Adjustments as for Model 1 plus adjustments for triglycerides, HDL cholesterol, brachial SBP and DBP (instead of PP), and waist circumference. Model 3: Adjustments as for Model 1 plus adjustments for triglycerides, HDL cholesterol, brachial SBP and DBP (instead of PP), and BMI. * BP in Model 1 is brachial pulse pressure, in Model 2 is brachial systolic BP, and in Model 3 is brachial systolic BP. BMI, body mass index; BP, blood pressure; Concentricity, increased left ventricular relative wall thickness; Decr, decreased; GFR, estimated glomerular filtration rate; Incr, increased; HOMA-IR, homeostatic model assessment for insulin resistance; LVH-BSA, increased left ventricular mass indexed to body surface area; PWV, pulse wave velocity; QUICKI, quantitative insulin sensitivity check index; WC, waist circumference.

## Data Availability

All relevant data are contained within this manuscript.

## Supplementary Materials

The following supporting information can be downloaded at: https://www.mdpi.com/article/10.3390/jcm14082703/s1.
