# Supplementary material for: Differential Association Between Ten Indices of Insulin Resistance and End-Organ Damage in a Community of African Ancestry in Africa
Source: J Clin Med. 2025 Apr 15;14(8):2703. doi: 10.3390/jcm14082703 (PMC12027772; doi:10.3390/jcm14082703)
Supplement: Supplementary file 1 [file jcm-14-02703-s001.zip › jcm-3551047-supplementary.pdf]

On-line Supplement

Differential Association Between Ten Indices of Insulin Resistance and End-Organ Damage in a Community of African Ancestry in Africa.

Angela J Woodiwiss, Gavin R Norton, Carlos D Libhaber, Pinhas Sareli, Patrick HC Dessein.

From the Cardiovascular Pathophysiology and Genomics Research Unit, Department of Physiology, School of Biomedical Sciences, Faculty of Health Sciences, University of the Witwatersrand, Johannesburg, South Africa.

Running title: IR indices and end-organ damage

Conflicts of interest: None

This study was supported by the Medical Research Council of South Africa, the University Research Council of the University of the Witwatersrand, the South African National Research Foundation, and the Circulatory Disorders Research Trust.

Correspondence and reprint requests: Angela J Woodiwiss: Cardiovascular Pathophysiology and Genomics Research Unit, Department of Physiology, School of Biomedical Sciences, University of the Witwatersrand Medical School, 7 York Road, Parktown, 2193, Johannesburg, South Africa. Tel: +27 11 717 2363, e-mail: angela.woodiwiss@wits.ac.za

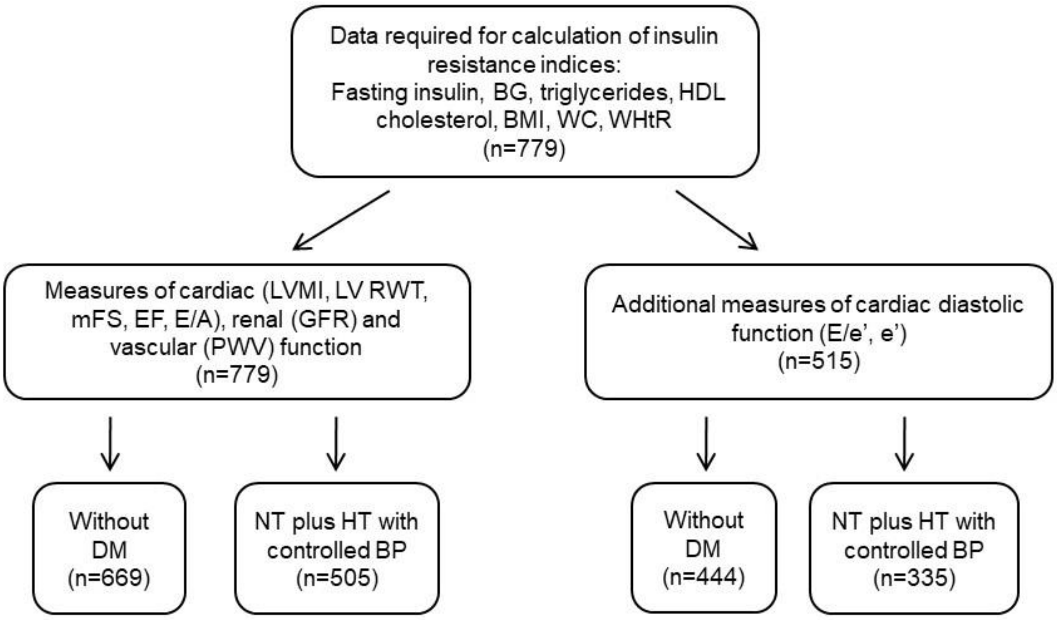

Figure S1. Flow chart of the study sample sizes and study sub-groups. BG, blood glucose; BMI, body mass index; BP, blood pressure; DM, diabetes mellitus; E/A, early-to-late mitral velocity; e', myocardial tissue lengthening in early diastole at the mitral annulus; E/e', transmitral early blood flow velocity/velocity of the mean value of lateral and septal wall myocardial tissue lengthening in early diastole at the mitral annulus; EF, ejection fraction; GFR, estimated glomerular filtration rate; HDL, high-density lipoprotein; HT, hypertension; LVMI, left ventricular mass indexed to body surface area; LV RWT, left ventricular relative wall thickness; mFS, left ventricular midwall fractional shortening; NT, normotension; PWV, pulse wave velocity; WC, waist circumference; WHtR, waist-to-height ratio.

Table S1. Characteristics of the normotensive participants (NT) plus the hypertensive (HT) participants with controlled blood pressure (BP) (n=505).

	NT + HT with controlled BP
Sample size	505
% Female (n)	67.9 (343)
Age (years)	39.7±17.1***
% <65 years of age (n)	81.8 (450)
Body mass index (kg/m^2)	28.1±7.3*
Waist circumference (cm)	88.7±16.5**
% Overweight (n)	26.7 (135)
% Obese (n)	34.8 (176)*
Brachial SBP (mm Hg)	116±11***
Brachial DBP (mm Hg)	76±7***
% Hypertension (n)	17.8 (90)***
% Uncontrolled BP (n)	0 (0)
% HT with uncontrolled BP (n)	0 (0)
% Treated for hypertension (n)	17.8 (90)**
% HT treated for HT (n)	100 (90/90)**
Heart rate (beats/min)	65.8±11.9
% Regular tobacco intake (n)	14.3 (72)
% Regular alcohol intake (n)	20.0 (101)
% Diabetes mellitus (n)	10.7 (54)
<u>Fasting plasma concentrations</u>	
Glucose (mmol/l)	4.50 (4.20 to 5.00)
Glycated haemoglobin (%)	5.72 (5.50 to 6.00)
Insulin (μU/ml)	7.36 (3.96 to 13.35)
Total cholesterol (mmol/l)	4.50 (4.00 to 5.16)
LDL cholesterol (mmol/l)	2.57 (2.15 to 3.10)
HDL cholesterol (mmol/l)	1.40 (1.20 to 1.50)
Triglycerides (mmol/l)	1.00 (0.70 to 1.40)
<u>Insulin resistance indices</u>	
HOMA-IR	1.56 (0.82 to 2.95)
QUICKI	0.36±0.05
METS-IR	40.1±12.1*
TyG	8.25±0.63**
TyG-BMI	232.9±67.3**
TyG-WC	735.6±167.5***
TyG-WHtR	4.56±1.08***
TyG-HDL	1.58 (1.14 to 2.43)
LAP	25.2 (11.7 to 50.2)*
VAI	1.14 (0.76 to 1.83)
<u>End organ measures</u>	
LVMI (g/m^2)	70.6±27.7**
% LV hypertrophy (n)	11.3 (57)*
LV relative wall thickness	0.36±0.07**
% Concentricity of LV	17.8 (90)
LV midwall fractional shortening	0.27±0.07*
EF (%)	67.1±8.1

E/A	1.42±0.50***
E/e' (n)	6.67±3.38 (335)**
e' (n)	12.55±3.93 (335)***
GFR (ml/min per 1.73m^2)	102.6±19.99***
% Decreased GFR (n)	14.6 (74)***
Pulse wave velocity (m/s)	5.36±1.92***
% Increased PWV (n)	8.3 (42)***

Data are shown as mean ± SD, median (interquartile range), or percentages. BP, blood pressure; DBP, diastolic blood pressure; E/A, early-to-late mitral velocity; e', myocardial tissue lengthening in early diastole at the mitral annulus; E/e', transmitral early blood flow velocity/velocity of the mean value of lateral and septal wall myocardial tissue lengthening in early diastole at the mitral annulus; EF, ejection fraction; GFR, estimated glomerular filtration rate; HDL, high-density lipoprotein; HOMA-IR, homeostatic model assessment for insulin resistance; HT, hypertension; LAP, lipid accumulation product; LDL, low-density lipoprotein; LV, left ventricle; LVMI, left ventricular mass indexed to body surface area; METS-IR, metabolic score for insulin resistance; PWV, pulse wave velocity; QUICKI, quantitative insulin sensitivity check index; SBP, systolic blood pressure; TyG, triglyceride–glucose index; TyG-BMI, triglyceride–body mass index; TyG-HDL, triglycerides to high-density cholesterol concentrations; TyG-WC, triglyceride–waist circumference; TyG-WHtR, triglyceride–waist-to-height ratio; VAI, visceral adiposity index. *p<0.05, **p<0.005, ***p<0.0001 versus all the participants.

Table S2. Unadjusted relationships between various indices of insulin resistance and end-organ measures in the participants without DM (n=669).

	r (95%CI)	p-value	r (95%CI)	p-value	r (95%CI)	p-value
	**LVMI**		**LV RWT**		**EF**	
lnHOMA-IR	0.147 (0.072 to 0.221)	**=0.0001**	0.199 (0.125 to 0.271)	**<0.0001**	-0.006 (-0.081 to 0.070)	=0.8837
QUICKI	-0.112 (-0.186 to -0.036)	**=0.0037**	-0.171 (-0.244 to -0.097)	**<0.0001**	0.011 (-0.065 to 0.086)	=0.7864
METS-IR	0.069 (-0.007 to 0.144)	=0.0748	0.158 (0.083 to 0.231)	**<0.0001**	-0.010 (-0.085 to 0.066)	=0.8022
TyG	0.128 (0.052 to 0.201)	**=0.0009**	0.150 (0.075 to 0.223)	**<0.0001**	0.012 (-0.063 to 0.088)	=0.7475
TyG-BMI	0.073 (-0.002 to 0.148)	=0.0574	0.153 (0.078 to 0.226)	**<0.0001**	-0.005 (-0.081 to 0.070)	=0.8874
TyG-WC	0.141 (0.066 to 0.214)	**=0.0003**	0.160 (0.085 to 0.233)	**<0.0001**	-0.007 (-0.082 to 0.069)	=0.8625
TyG-WHtR	0.137 (0.062 to 0.211)	**=0.0004**	0.181 (0.107 to 0.254)	**<0.0001**	0.007 (-0.069 to 0.083)	=0.8543
lnTyG-HDL	0.083 (0.007 to 0.158)	**=0.0316**	0.097 (0.021 to 0.171)	**=0.0122**	0.024 (-0.052 to 0.099)	=0.5391
lnLAP	0.092 (0.015 to 0.167)	**=0.0188**	0.143 (0.068 to 0.217)	**=0.0002**	0.004 (-0.072 to 0.081)	=0.9109
lnVAI	0.056 (-0.020 to 0.131)	=0.1464	0.115 (0.039 to 0.189)	**=0.0029**	0.030 (-0.046 to 0.105)	=0.4437
	**mFS**		**E/A**		**E/e' (n=444)**	
lnHOMA-IR	-0.051 (-0.126 to 0.025)	=0.1900	-0.139 (-0.213 to -0.064)	**=0.0003**	0.144 (0.052 to 0.234)	**=0.0023**
QUICKI	0.030 (-0.046 to 0.105)	=0.4426	0.123 (0.047 to 0.197)	**=0.0014**	-0.147 (-0.237 to -0.055)	**=0.0018**
METS-IR	-0.029 (-0.105 to 0.047)	=0.4483	-0.389 (-0.451 to -0.322)	**<0.0001**	0.287 (0.199 to 0.370)	**<0.0001**
TyG	-0.028 (-0.103 to 0.048)	=0.4778	-0.384 (-0.441 to -0.311)	**<0.0001**	0.137 (0.045 to 0.227)	**=0.0037**
TyG-BMI	-0.044 (-0.119 to 0.032)	=0.2601	-0.420 (-0.480 to -0.355)	**<0.0001**	0.303 (0.216 to 0.385)	**<0.0001**
TyG-WC	-0.063 (-0.138 to 0.013)	=0.1020	-0.490 (-0.546 to -0.430)	**<0.0001**	0.287 (0.199 to 0.370)	**<0.0001**
TyG-WHtR	-0.058 (-0.133 to 0.018)	=0.1336	-0.505 (-0.559 to -0.446)	**<0.0001**	0.327 (0.241 to 0.407)	**<0.0001**
lnTyG-HDL	0.024 (-0.052 to 0.099)	=0.5406	-0.280 (-0.348 to -0.209)	**<0.0001**	0.102 (0.009 to 0.193)	**=0.0319**
lnLAP	-0.066 (-0.142 to 0.010)	=0.0889	-0.470 (-0.527 to -0.408)	**<0.0001**	0.257 (0.167 to 0.342)	**<0.0001**
lnVAI	0.012 (-0.064 to 0.088)	=0.7505	-0.341 (-0.406 to -0.272)	**<0.0001**	0.163 (0.071 to 0.252)	**=0.0005**
	**e' (n=444)**		**PWV**		**GFR**	
lnHOMA-IR	-0.131 (-0.221 to -0.038)	**=0.0058**	0.081 (0.005 to 0.156)	**=0.0363**	-0.214 (-0.285 to -0.140)	**<0.0001**
QUICKI	0.115 (0.022 to 0.205)	**=0.0156**	-0.048 (-0.123 to 0.028)	=0.2169	0.191 (0.117 to 0.263)	**<0.0001**

METS-IR	-0.410 (-0.484 to -0.329)	**<0.0001**	0.237 (0.164 to 0.307)	**<0.0001**	-0.319 (-0.385 to -0.249)	**<0.0001**
TyG	-0.382 (-0.459 to -0.299)	**<0.0001**	0.307 (0.237 to 0.374)	**<0.0001**	-0.342 (-0.407 to -0.273)	**<0.0001**
TyG-BMI	-0.438 (-0.410 to -0.359)	**<0.0001**	0.270 (0.198 to 0.338)	**<0.0001**	-0.344 (-0.409 to -0.275)	**<0.0001**
TyG-WC	-0.488 (-0.556 to -0.414)	**<0.0001**	0.367 (0.299 to 0.431)	**<0.0001**	-0.394 (-0.456 to -0.328)	**<0.0001**
TyG-WHtR	-0.512 (-0.577 to -0.439)	**<0.0001**	0.377 (0.309 to 0.439)	**<0.0001**	-0.399 (-0.461 to -0.333)	**<0.0001**
lnTyG-HDL	-0.311 (-0.392 to -0.224)	**<0.0001**	0.215 (0.142 to 0.286)	**<0.0001**	-0.244 (-0.314 to -0.171)	**<0.0001**
lnLAP	-0.454 (-0.525 to -0.375)	**<0.0001**	0.358 (0.290 to 0.423)	**<0.0001**	-0.410 (-0.471 to -0.344)	**<0.0001**
lnVAI	-0.356 (-0.435 to -0.272)	**<0.0001**	0.241 (0.168 to 0.310)	**<0.0001**	-0.289 (-0.357 to -0.218)	**<0.0001**

All significant associations are shown in bold type. CI, confidence interval; E/A, early-to-late mitral velocity; e’, myocardial tissue lengthening in early diastole at the mitral annulus; E/e’, transmitral early blood flow velocity/velocity of the mean value of lateral and septal wall myocardial tissue lengthening in early diastole at the mitral annulus; EF, ejection fraction; GFR, estimated glomerular filtration rate; lnHOMA-IR, natural logarithm of homeostatic model assessment for insulin resistance; lnLAP, natural logarithm of lipid accumulation product; LVMI, left ventricular mass indexed to body surface area; LV RWT, left ventricular relative wall thickness; METS-IR, metabolic score for insulin resistance; mFS, left ventricular midwall fractional shortening; PWV, pulse wave velocity; QUICKI, quantitative insulin sensitivity check index; r, Pearson’s correlation coefficient; TyG, triglyceride–glucose index; TyG-BMI, triglyceride–body mass index; lnTyG-HDL, natural logarithm of triglycerides to high-density cholesterol concentrations; TyG-WC, triglyceride–waist circumference; TyG-WHtR, triglyceride–waist-to-height ratio; lnVAI, natural logarithm of visceral adiposity index.

Table S3. Unadjusted relationships between various indices of insulin resistance and end-organ measures in the normotensive (NT) participants and the hypertensive (HT) participants with controlled blood pressure (BP) (n=505)**.**

	r (95%CI)	p-value	r (95%CI)	p-value	r (95%CI)	p-value
	**LVMI**		**LV RWT**		**EF**	
lnHOMA-IR	0.157 (0.071 to 0.241)	**=0.0004**	0.181 (0.096 to 0.264)	**<0.0001**	-0.040 (-0.127 to 0.047)	=0.3677
QUICKI	-0.126 (-0.211 to -0.039)	**=0.0044**	-0.157 (-0.241 to -0.071)	**=0.0004**	0.040 (-0.047 to 0.127)	=0.3658
METS-IR	-0.011 (-0.098 to 0.077)	=0.8113	0.077 (-0.010 to 0.163)	=0.0823	-0.058 (-0.145 to 0.029)	=0.1916
TyG	0.084 (-0.003 to 0.170)	=0.0581	0.127 (0.040 to 0.212)	**=0.0041**	-0.014 (-0.101 to 0.073)	=0.7482
TyG-BMI	-0.001 (-0.087 to 0.087)	=0.9996	0.069 (-0.018 to 0.155)	=0.1200	-0.056 (-0.142 to 0.032)	=0.2095
TyG-WC	0.043 (-0.045 to 0.129)	=0.3394	0.081 (-0.007 to 0.167)	=0.0698	-0.045 (-0.132 to 0.042)	=0.3075
TyG-WHtR	0.033 (-0.054 to 0.120)	=0.4600	0.114 (0.027 to 0.199)	**=0.0105**	-0.026 (-0.113 to 0.061)	=0.5614
lnTyG-HDL	0.033 (-0.054 to 0.120)	=0.4603	0.076 (-0.011 to 0.162)	=0.0863	0.006 (-0.081 to 0.093)	=0.8897
lnLAP	0.002 (-0.085 to 0.090)	=0.9564	0.075 (-0.013 to 0.161)	=0.0961	-0.028 (-0.115 to 0.060)	=0.5394
lnVAI	-0.017 (-0.104 to 0.070)	=0.7027	0.084 (-0.003 to 0.170)	=0.0596	0.007 (-0.081 to 0.094)	=0.8820
	**mFS**		**E/A**		**E/e' (n=336)**	
lnHOMA-IR	-0.073 (-0.159 to 0.014)	=0.1012	-0.191 (-0.273 to -0.105)	**<0.0001**	0.190 (0.085 to 0.291)	**=0.0004**
QUICKI	0.051 (-0.036 to 0.138)	=0.2523	0.174 (0.088 to 0.257)	**<0.0001**	-0.187 (-0.288 to -0.081)	**=0.0006**
METS-IR	-0.040 (-0.127 to 0.047)	=0.3662	-0.353 (-0.427 to -0.274)	**<0.0001**	0.305 (0.204 to 0.399)	**<0.0001**
TyG	-0.083 (-0.169 to 0.004)	=0.0621	-0.324 (-0.400 to -0.244)	**<0.0001**	0.182 (0.076 to 0.284)	**=0.0008**
TyG-BMI	-0.068 (-0.154 to 0.019)	=0.1266	-0.370 (-0.443 to -0.292)	**<0.0001**	0.321 (0.221 to 0.413)	**<0.0001**
TyG-WC	-0.079 (-0.165 to 0.008)	=0.0763	-0.409 (-0.479 to -0.333)	**<0.0001**	0.300 (0.198 to 0.394)	**<0.0001**
TyG-WHtR	-0.081 (-0.167 to 0.006)	=0.0685	-0.428 (-0.497 to -0.354)	**<0.0001**	0.333 (0.234 to 0.424)	**<0.0001**
lnTyG-HDL	-0.011 (-0.098 to 0.077)	=0.8113	-0.261 (-0.340 to -0.177)	**<0.0001**	0.129 (0.022 to 0.233)	**=0.0178**
lnLAP	-0.099 (-0.185 to -0.011)	**=0.0276**	-0.386 (-0.458 to -0.309)	**<0.0001**	0.248 (0.143 to 0.347)	**<0.0001**
lnVAI	-0.028 (-0.115 to 0.059)	=0.5325	-0.313 (-0.390 to -0.232)	**<0.0001**	0.182 (0.076 to 0.284)	**=0.0008**
	**e' (n=336)**		**PWV**		**GFR**	
lnHOMA-IR	-0.263 (-0.359 to -0.160)	**<0.0001**	0.205 (0.120 to 0.287)	**<0.0001**	-0.239 (-0.319 to -0.154)	**<0.0001**
QUICKI	0.249 (0.145 to 0.347)	**<0.0001**	-0.187 (-0.270 to -0.101)	**<0.0001**	0.208 (0.123 to 0.290)	**<0.0001**

METS-IR	-0.485 (-0.563 to -0.398)	**<0.0001**	0.362 (0.283 to 0.435)	**<0.0001**	-0.342 (-0.417 to -0.262)	**<0.0001**
TyG	-0.451 (-0.531 to -0.360)	**<0.0001**	0.415 (0.340 to 0.484)	**<0.0001**	-0.336 (-0.411 to -0.256)	**<0.0001**
TyG-BMI	-0.507 (-0.582 to -0.422)	**<0.0001**	0.385 (0.307 to 0.456)	**<0.0001**	-0.364 (-0.437 to -0.286)	**<0.0001**
TyG-WC	-0.552 (-0.622 to -0.472)	**<0.0001**	0.471 (0.399 to 0.536)	**<0.0001**	-0.388 (-0.459 to -0.310)	**<0.0001**
TyG-WHtR	-0.573 (-0.640 to -0.496)	**<0.0001**	0.468 (0.397 to 0.533)	**<0.0001**	-0.400 (-0.470 to -0.324)	**<0.0001**
lnTyG-HDL	-0.386 (-0.473 to -0.290)	**<0.0001**	0.283 (0.200 to 0.361)	**<0.0001**	-0.246 (-0.326 to -0.162)	**<0.0001**
lnLAP	-0.487 (-0.565 to -0.399)	**<0.0001**	0.428 (0.353 to 0.497)	**<0.0001**	-0.391 (-0.462 to -0.313)	**<0.0001**
lnVAI	-0.437 (-0.520 to -0.346)	**<0.0001**	0.320 (0.239 to 0.396)	**<0.0001**	-0.291 (-0.369 to -0.209)	**<0.0001**

All significant associations are shown in bold type. CI, confidence interval; E/A, early-to-late mitral velocity; e', myocardial tissue lengthening in early diastole at the mitral annulus; E/e', transmitral early blood flow velocity/velocity of the mean value of lateral and septal wall myocardial tissue lengthening in early diastole at the mitral annulus; EF, ejection fraction; GFR, estimated glomerular filtration rate; lnHOMA-IR, natural logarithm of homeostatic model assessment for insulin resistance; lnLAP, natural logarithm of lipid accumulation product; LVMI, left ventricular mass indexed to body surface area; LV RWT, left ventricular relative wall thickness; METS-IR, metabolic score for insulin resistance; mFS, left ventricular midwall fractional shortening; PWV, pulse wave velocity; QUICKI, quantitative insulin sensitivity check index; r, Pearson's correlation coefficient; TyG, triglyceride–glucose index; TyG-BMI, triglyceride–body mass index; lnTyG-HDL, natural logarithm of triglycerides to high-density cholesterol concentrations; TyG-WC, triglyceride–waist circumference; TyG-WHtR, triglyceride–waist-to-height ratio; lnVAI, natural logarithm of visceral adiposity index.

Table S4. Unadjusted relationships between various indices of insulin resistance and end-organ measures in the female participants (n=508).

	r (95%CI)	p-value	r (95%CI)	p-value	r (95%CI)	p-value
	**LVMI**		**LV RWT**		**EF**	
lnHOMA-IR	0.154 (0.067 to 0.237)	**=0.0005**	0.172 (0.087 to 0.255)	**<0.0001**	0.028 (-0.059 to 0.115)	=0.5249
QUICKI	-0.134 (-0.218 to -0.047)	**=0.0024**	-0.155 (-0.238 to -0.069)	**=0.0005**	-0.020 (-0.107 to 0.067)	=0.6463
METS-IR	0.130 (0.043 to 0.215)	**=0.0033**	0.148 (0.062 to 0.232)	**=0.0008**	0.001 (-0.088 to 0.086)	=0.9820
TyG	0.128 (0.041 to 0.212)	**=0.0039**	0.195 (0.110 to 0.277)	**<0.0001**	0.072 (-0.015 to 0.158)	=0.1059
TyG-BMI	0.162 (0.076 to 0.245)	**=0.0002**	0.153 (0.067 to 0.237)	**=0.0005**	0.005 (-0.082 to 0.092)	=0.9049
TyG-WC	0.180 (0.095 to 0.263)	**<0.0001**	0.162 (0.076 to 0.246)	**=0.0002**	0.021 (-0.066 to 0.107)	=0.6411
TyG-WHtR	0.206 (0.121 to 0.288)	**<0.0001**	0.194 (0.109 to 0.276)	**<0.0001**	0.031 (-0.056 to 0.117)	=0.4892
lnTyG-HDL	0.056 (-0.031 to 0.142)	=0.2086	0.128 (0.042 to 0.213)	**=0.0038**	0.036 (-0.051 to 0.123)	=0.4182
lnLAP	0.139 (0.052 to 0.223)	**=0.0018**	0.154 (0.067 to 0.237)	**=0.0005**	0.024 (-0.064 to 0.111)	=0.5927
lnVAI	0.069 (-0.019 to 0.155)	=0.1230	0.129 (0.043 to 0.214)	**=0.0035**	0.037 (-0.051 to 0.123)	=0.4098
	**mFS**		**E/A**		**E/e' (n=336)**	
lnHOMA-IR	-0.016 (-0.102 to 0.071)	=0.7256	-0.196 (-0.278 to -0.110)	**<0.0001**	0.145 (0.038 to 0.248)	**=0.0078**
QUICKI	0.005 (-0.082 to 0.092)	=0.9022	0.186 (0.100 to 0.268)	**<0.0001**	-0.151 (-0.253 to -0.044)	**=0.0056**
METS-IR	0.014 (-0.073 to 0.101)	=0.7499	-0.396 (-0.467 to -0.320)	**<0.0001**	0.243 (0.139 to 0.341)	**<0.0001**
TyG	-0.025 (-0.111 to 0.063)	=0.5801	-0.403 (-0.473 to -0.327)	**<0.0001**	0.212 (0.107 to 0.312)	**<0.0001**
TyG-BMI	-0.008 (-0.095 to 0.079)	=0.8560	-0.426 (-0.494 to -0.351)	**<0.0001**	0.258 (0.154 to 0.354)	**<0.0001**
TyG-WC	-0.013 (-0.100 to 0.074)	=0.7664	-0.473 (-0.537 to -0.402)	**<0.0001**	0.277 (0.174 to 0.372)	**<0.0001**
TyG-WHtR	-0.024 (-0.111 to 0.063)	=0.5926	-0.496 (-0.559 to -0.427)	**<0.0001**	0.302 (0.201 to 0.395)	**<0.0001**
lnTyG-HDL	0.016 (-0.071 to 0.103)	=0.7242	-0.343 (-0.418 to -0.264)	**<0.0001**	0.160 (0.054 to 0.262)	**=0.0032**
lnLAP	-0.046 (-0.133 to 0.041)	=0.2979	-0.466 (-0.531 to -0.394)	**<0.0001**	0.255 (0.151 to 0.352)	**<0.0001**
lnVAI	0.012 (-0.075 to 0.099)	=0.7834	-0.362 (-0.435 to -0.283)	**<0.0001**	0.174 (0.068 to 0.275)	**=0.0013**
	**e' (n=336)**		**PWV**		**GFR**	
lnHOMA-IR	-0.214 (-0.314 to -0.109)	**<0.0001**	0.197 (0.111 to 0.279)	**<0.0001**	-0.225 (-0.306 to -0.140)	**<0.0001**
QUICKI	0.218 (0.114 to 0.318)	**<0.0001**	-0.187 (-0.269 to -0.101)	**<0.0001**	0.205 (0.120 to 0.287)	**<0.0001**

METS-IR	-0.479 (-0.557 to -0.392)	**<0.0001**	0.298 (0.217 to 0.375)	**<0.0001**	-0.296 (-0.373 to -0.214)	**<0.0001**
TyG	-0.491 (-0.567 to -0.404)	**<0.0001**	0.411 (0.335 to 0.480)	**<0.0001**	-0.362 (-0.435 to -0.284)	**<0.0001**
TyG-BMI	-0.504 (-0.580 to -0.419)	**<0.0001**	0.338 (0.258 to 0.412)	**<0.0001**	-0.328 (-0.403 to -0.248)	**<0.0001**
TyG-WC	-0.561 (-0.629 to -0.482)	**<0.0001**	0.411 (0.335 to 0.480)	**<0.0001**	-0.371 (-0.443 to -0.293)	**<0.0001**
TyG-WHtR	-0.585 (-0.651 to -0.509)	**<0.0001**	0.434 (0.361 to 0.502)	**<0.0001**	-0.383 (-0.454 to -0.306)	**<0.0001**
lnTyG-HDL	-0.438 (-0.520 to -0.346)	**<0.0001**	0.283 (0.201 to 0.361)	**<0.0001**	-0.276 (-0.354 to -0.194)	**<0.0001**
lnLAP	-0.530 (-0.603 to -0.448)	**<0.0001**	0.403 (0.327 to 0.473)	**<0.0001**	-0.380 (-0.452 to -0.303)	**<0.0001**
lnVAI	-0.458 (-0.538 to -0.369)	**<0.0001**	0.308 (0.226 to 0.384)	**<0.0001**	-0.291 (-0.368 to -0.209)	**<0.0001**

All significant associations are shown in bold type. CI, confidence interval; E/A, early-to-late mitral velocity; e’, myocardial tissue lengthening in early diastole at the mitral annulus; E/e’, transmitral early blood flow velocity/velocity of the mean value of lateral and septal wall myocardial tissue lengthening in early diastole at the mitral annulus; EF, ejection fraction; GFR, estimated glomerular filtration rate; lnHOMA-IR, natural logarithm of homeostatic model assessment for insulin resistance; lnLAP, natural logarithm of lipid accumulation product; LVMI, left ventricular mass indexed to body surface area; LV RWT, left ventricular relative wall thickness; METS-IR, metabolic score for insulin resistance; mFS, left ventricular midwall fractional shortening; PWV, pulse wave velocity; QUICKI, quantitative insulin sensitivity check index; r, Pearson’s correlation coefficient; TyG, triglyceride–glucose index; TyG-BMI, triglyceride–body mass index; lnTyG-HDL, natural logarithm of triglycerides to high-density cholesterol concentrations; TyG-WC, triglyceride–waist circumference; TyG-WHtR, triglyceride–waist-to-height ratio; lnVAI, natural logarithm of visceral adiposity index.

Table S5. Unadjusted relationships between various indices of insulin resistance and end-organ measures in the male participants (n=271).

	r (95%CI)	p-value	r (95%CI)	p-value	r (95%CI)	p-value
	**LVMI**		**LV RWT**		**EF**	
lnHOMA-IR	0.147 (0.028 to 0.261)	**=0.0155**	0.251 (0.136 to 0.359)	**<0.0001**	-0.048 (-0.166 to 0.072)	=0.4344
QUICKI	-0.114 (-0.230 to 0.005)	=0.0604	-0.218 (-0.328 to -0.101)	**=0.0003**	0.053 (-0.066 to 0.171)	=0.3821
METS-IR	0.149 (0.030 to 0.263)	**=0.0143**	0.213 (0.096 to 0.323)	**=0.0004**	-0.070 (-0.187 to 0.050)	=0.2520
TyG	0.091 (-0.029 to 0.207)	=0.1373	0.163 (0.044 to 0.277)	**=0.0071**	-0.048 (-0.166 to 0.072)	=0.4325
TyG-BMI	0.162 (0.043 to 0.275)	**=0.0075**	0.218 (0.101 to 0.328)	**=0.0003**	-0.077 (-0.194 to 0.043)	=0.2087
TyG-WC	0.178 (0.060 to 0.291)	**=0.0032**	0.214 (0.096 to 0.324)	**=0.0004**	-0.074 (-0.191 to 0.046)	=0.2256
TyG-WHtR	0.199 (0.081 to 0.310)	**=0.0010**	0.233 (0.116 to 0.342)	**=0.0001**	-0.068 (-0.186 to 0.051)	=0.2630
lnTyG-HDL	0.071 (-0.049 to 0.188)	=0.2475	0.115 (-0.005 to 0.230)	=0.0594	0.025 (-0.094 to 0.144)	=0.6785
lnLAP	0.154 (0.033 to 0.269)	**=0.0124**	0.170 (0.050 to 0.285)	**=0.0055**	-0.036 (-0.156 to 0.085)	=0.5622
lnVAI	0.084 (-0.036 to 0.201)	=0.1700	0.123 (0.004 to 0.238)	**=0.0431**	0.020 (-0.099 to 0.139)	=0.7373
	**mFS**		**E/A**		**E/e’ (n=180)**	
lnHOMA-IR	-0.115 (-0.231 to 0.004)	=0.0582	-0.191 (-0.303 to -0.073)	**=0.0015**	0.268 (0.126 to 0.398)	**=0.0003**
QUICKI	0.080 (-0.040 to 0.197)	=0.1900	0.158 (0.039 to 0.272)	**=0.0090**	-0.241 (-0.374 to -0.098)	**=0.0011**
METS-IR	-0.078 (-0.195 to 0.042)	=0.2017	-0.352 (-0.451 to -0.242)	**<0.0001**	0.273 (0.132 to 0.403)	**=0.0002**
TyG	-0.078 (-0.195 to 0.042)	=0.1997	-0.391 (-0.487 to -0.284)	**<0.0001**	0.194 (0.048 to 0.330)	**=0.0090**
TyG-BMI	-0.116 (-0.232 to 0.003)	=0.0559	-0.395 (-0.490 to -0.288)	**<0.0001**	0.295 (0.155 to 0.422)	**<0.0001**
TyG-WC	-0.135 (-0.250 to -0.016)	**=0.0263**	-0.484 (-0.570 to -0.387)	**<0.0001**	0.255 (0.112 to 0.386)	**=0.0005**
TyG-WHtR	-0.132 (-0.247 to -0.013)	**=0.0301**	-0.512 (-0.594 to -0.417)	**<0.0001**	0.303 (0.163 to 0.430)	**<0.0001**
lnTyG-HDL	0.029 (-0.090 to 0.148)	=0.6301	-0.300 (-0.404 to -0.187)	**<0.0001**	0.139 (-0.007 to 0.279)	=0.0621
lnLAP	-0.089 (-0.208 to 0.032)	=0.1471	-0.464 (-0.553 to -0.363)	**<0.0001**	0.213 (0.066 to 0.350)	**=0.0047**
lnVAI	0.014 (-0.105 to 0.133)	=0.8153	-0.332 (-0.434 to -0.221)	**<0.0001**	0.143 (-0.004 to 0.283)	=0.0556
	**e’ (n=180)**		**PWV**		**GFR**	
lnHOMA-IR	-0.225 (-0.359 to -0.081)	**=0.0023**	0.086 (-0.033 to 0.203)	=0.1566	-0.290 (-0.395 to -0.176)	**<0.0001**
QUICKI	0.175 (0.029 to 0.313)	**=0.0187**	-0.053 (-0.171 to 0.067)	=0.3849	0.268 (0.154 to 0.375)	**<0.0001**

METS-IR	-0.359 (-0.479 to -0.224)	**<0.0001**	0.220 (0.103 to 0.330)	**=0.0002**	-0.371 (-0.468 to -0.262)	**<0.0001**
TyG	-0.387 (-0.504 to -0.254)	**<0.0001**	0.275 (0.161 to 0.382)	**<0.0001**	-0.348 (-0.448 to -0.238)	**<0.0001**
TyG-BMI	-0.411 (-0.525 to -0.281)	**<0.0001**	0.257 (0.142 to 0.365)	**<0.0001**	-0.398 (-0.493 to -0.292)	**<0.0001**
TyG-WC	-0.448 (-0.557 to -0.322)	**<0.0001**	0.354 (0.244 to 0.453)	**<0.0001**	-0.459 (-0.547 to -0.359)	**<0.0001**
TyG-WHtR	-0.489 (-0.592 to -0.368)	**<0.0001**	0.374 (0.266 to 0.472)	**<0.0001**	-0.480 (-0.566 to -0.382)	**<0.0001**
lnTyG-HDL	-0.310 (-0.436 to -0.171)	**<0.0001**	0.187 (0.069 to 0.299)	**=0.0020**	-0.272 (-0.378 to -0.157)	**<0.0001**
lnLAP	-0.449 (-0.560 to -0.321)	**<0.0001**	0.354 (0.244 to 0.455)	**<0.0001**	-0.461 (-0.550 to -0.359)	**<0.0001**
lnVAI	-0.328 (-0.452 to -0.190)	**<0.0001**	0.218 (0.101 to 0.328)	**=0.0003**	-0.301 (-0.405 to -0.188)	**<0.0001**

All significant associations are shown in bold type. CI, confidence interval; E/A, early-to-late mitral velocity; e’, myocardial tissue lengthening in early diastole at the mitral annulus; E/e’, transmitral early blood flow velocity/velocity of the mean value of lateral and septal wall myocardial tissue lengthening in early diastole at the mitral annulus; EF, ejection fraction; GFR, estimated glomerular filtration rate; lnHOMA-IR, natural logarithm of homeostatic model assessment for insulin resistance; lnLAP, natural logarithm of lipid accumulation product; LVMI, left ventricular mass indexed to body surface area; LV RWT, left ventricular relative wall thickness; METS-IR, metabolic score for insulin resistance; mFS, left ventricular midwall fractional shortening; PWV, pulse wave velocity; QUICKI, quantitative insulin sensitivity check index; r, Pearson’s correlation coefficient; TyG, triglyceride–glucose index; TyG-BMI, triglyceride–body mass index; lnTyG-HDL, natural logarithm of triglycerides to high-density cholesterol concentrations; TyG-WC, triglyceride–waist circumference; TyG-WHtR, triglyceride–waist-to-height ratio; lnVAI, natural logarithm of visceral adiposity index.

Table S6. Adjusted relationships between HOMA-IR, insulin and blood glucose (BG), and end-organ measures (n=779). Model 1 = HOMA-IR plus adjustors; Model 2 = insulin plus adjustors; Model 3 = BG plus adjustors; Model 4 = Insulin and BG in the same model together with adjustors.

	Stand. β±sem **LVMI**	p-value	Stand. β±sem **LV RWT**	p-value	Stand. β±sem **EF**	p-value
Model 1: lnHOMA-IR	0.1169±0.0338	**=0.0006**	0.1600±0.0351	**<0.0001**	0.0013±0.0368	=0.9718
Model 2: lnInsulin	0.1121±0.0333	**=0.0008**	0.1368±0.0346	**<0.0001**	0.0004±0.0362	=0.9914
Model 3: lnBG	0.0526±0.0364	=0.1487	0.1275±0.0377	**=0.0008**	0.0032±0.0394	=0.9357
Model 4: lnInsulin	0.1067±0.0340	**=0.0018**	0.1181±0.0352	**=0.0008**	-0.0002±0.0370	=0.9957
lnBG	0.0294±0.0369	=0.4263	0.1018±0.0382	**=0.0079**	0.0032±0.0402	=0.9361
	**mFS**		**E/A**		**E/e’**	
Model 1: lnHOMA-IR	-0.0434±0.0368	=0.2384	-0.0837±0.0282	**=0.0031**	0.1378±0.0453	=**0.0024**
Model 2: lnInsulin	-0.0303±0.0362	=0.4036	-0.0787±0.0277	**=0.0046**	0.1186±0.0446	**=0.0081**
Model 3: lnBG	-0.0618±0.0393	=0.1161	-0.0495±0.0302	=0.1022	0.1144±0.0503	**=0.0232**
Model 4: ln Insulin	-0.0197±0.0369	=0.5948	-0.0725±0.0283	**=0.0106**	0.1012±0.0456	**=0.0268**
lnBG	-0.0576±0.0401	=0.1520	-0.0337±0.0307	**=0.0307**	0.0896±0.0513	=0.0813
	**e’**		**PWV**		**GFR**	
Model 1: lnHOMA-IR	-0.1274±0.0430	**=0.0032**	0.0602±0.0286	**=0.0356**	-0.1387±0.0266	**<0.0001**
Model 2: lnInsulin	-0.1343±0.0378	**=0.0004**	0.0275±0.0281	=0.3279	-0.1349±0.0261	**<0.0001**
Model 3: lnBG	-0.1217±0.0427	**=0.0046**	0.1025±0.0303	**=0.0008**	-0.0547±0.0288	=0.0578

Model 4: lnInsulin	-0.1162±0.0386	**=0.0027**	0.0090±0.0285	=0.7528	-0.1300±0.0267	**<0.0001**
lnBG	-0.0931±0.0434	**=0.0325**	0.1005±0.0310	**=0.0012**	-0.0264±0.0290	=0.3620

All significant associations are shown in bold type. Adjustments are for age, sex, regular alcohol intake, regular tobacco intake, treatment for hypertension, heart rate, and brachial pulse pressure. E/A, early-to-late mitral blood flow velocity; e', myocardial tissue lengthening in early diastole at the mitral annulus; E/e', transmitral early blood flow velocity/velocity of the mean value of lateral and septal wall myocardial tissue lengthening in early diastole at the mitral annulus; EF, ejection fraction; GFR, estimated glomerular filtration rate; lnBG, natural logarithm of blood glucose; lnHOMA-IR, natural logarithm of homeostatic model assessment for insulin resistance; lnInsulin, natural logarithm of insulin; LVMI, left ventricular mass indexed to body surface area; LV RWT, left ventricular relative wall thickness; mFS, left ventricular midwall fractional shortening; PWV, pulse wave velocity; Stand. standardized.

Table S7. Relationships between various indices of insulin resistance and end-organ measures, independent of confounders, in the female participants (n=508).

	Partial r (95%CI)	p-value	Partial r (95%CI)	p-value	Partial r (95%CI)	p-value
	**LVMI**		**LV RWT**		**EF**	
lnHOMA-IR	0.111 (0.024 to 0.197)	**=0.0126**	0.135 (0.048 to 0.220)	**=0.0024**	-0.009 (-0.118 to 0.099)	=0.8672
QUICKI	-0.086 (-0.172 to 0.002)	=0.0546	-0.115 (-0.201 to -0.028)	**=0.0097**	0.013 (-0.095 to 0.121)	=0.8116
METS-IR	-0.033 (-0.120 to 0.055)	=0.4673	0.009 (-0.078 to 0.097)	=0.8348	-0.082 (-0.188 to 0.027)	=0.1413
TyG	-0.036 (-0.123 to 0.051)	=0.4172	0.051 (-0.037 to 0.138)	=0.2554	0.042 (-0.067 to 0.149)	=0.4515
TyG-BMI	-0.012 (-0.100 to 0.075)	=0.7823	-0.001 (-0.088 to 0.087)	=0.9941	-0.077 (-0.184 to 0.031)	=0.1630
TyG-WC	-0.006 (-0.094 to 0.081)	=0.8909	-0.017 (-0.104 to 0.071)	=0.7049	-0.018 (-0.126 to 0.090)	=0.7447
TyG-WHtR	0.019 (-0.068 to 0.107)	=0.6638	0.015 (-0.073 to 0.102)	=0.7410	-0.021 (-0.129 to 0.088)	=0.7113
lnTyG-HDL	-0.094 (-0.179 to -0.006)	**=0.0362**	-0.001 (-0.088 to 0.087)	=0.9827	-0.006 (-0.114 to 0.103)	=0.9155
lnLAP	-0.061 (-0.148 to 0.027)	=0.1731	-0.026 (-0.114 to 0.062)	=0.5606	-0.037 (-0.144 to 0.072)	=0.5087
lnVAI	-0.094 (-0.180 to -0.006)	**=0.0356**	-0.016 (-0.098 to 0.078)	=0.8184	0.008 (-0.101 to 0.116)	=0.8905
	**mFS**		**E/A**		**E/e’ (n=336)**	
lnHOMA-IR	-0.011 (-0.098 to 0.077)	=0.8088	-0.124 (-0.209 to -0.036)	**=0.0055**	0.073 (-0.036 to 0.180)	=0.1870
QUICKI	0.001 (-0.087 to 0.088)	=0.9915	0.111 (0.023 to 0.196)	**=0.0129**	-0.079 (-0.186 to 0.030)	=0.1543
METS-IR	0.035 (-0.052 to 0.123)	=0.4282	-0.151 (-0.235 to -0.064)	**=0.0007**	0.061 (-0.048 to 0.168)	=0.2746
TyG	-0.008 (-0.096 to 0.079)	=0.8517	-0.074 (-0.160 to 0.014)	=0.0980	-0.016 (-0.125 to 0.092)	=0.7673
TyG-BMI	0.011 (-0.077 to 0.098)	=0.8049	-0.153 (-0.237 to -0.066)	**=0.0006**	0.061 (-0.048 to 0.168)	=0.2700
TyG-WC	0.009 (-0.079 to 0.096)	=0.8476	-0.149 (-0.233 to -0.062)	**=0.0008**	0.044 (-0.064 to 0.152)	=0.4250
TyG-WHtR	-0.004 (-0.091 to 0.084)	=0.9354	-0.160 (-0.244 to -0.073)	**=0.0003**	0.070 (-0.039 to 0.177)	=0.2084
lnTyG-HDL	0.036 (-0.052 to 0.123)	=0.4260	-0.079 (-0.165 to 0.009)	=0.0785	-0.046 (-0.154 to 0.062)	=0.4029
lnLAP	-0.033 (-0.121 to 0.055)	=0.4602	-0.138 (-0.223 to -0.050)	**=0.0020**	0.026 (-0.083 to 0.134)	=0.6407
lnVAI	0.031 (-0.057 to 0.119)	=0.4874	-0.083 (-0.169 to 0.005)	=0.0647	-0.043 (-0.151 to 0.065)	=0.4341
	**e’ (n=336)**		**PWV**		**GFR**	
lnHOMA-IR	-0.137 (-0.242 to -0.029)	**=0.0128**	0.121 (0.033 to 0.206)	**=0.0068**	-0.171 (-0.285 to -0.140)	**=0.0001**
QUICKI	0.149 (0.041 to 0.253)	**=0.0070**	-0.105 (-0.191 to -0.018)	**=0.0182**	0.139 (0.052 to 0.223)	**=0.0018**

METS-IR	-0.273 (-0.370 to -0.169)	**<0.0001**	-0.009 (-0.096 to 0.079)	=0.8448	0.027 (-0.385 to -0.249)	=0.5469
TyG	-0.166 (-0.269 to -0.058)	**=0.0026**	0.092 (0.005 to 0.178)	**=0.0383**	-0.037 (-0.407 to -0.273)	=0.4137
TyG-BMI	-0.272 (-0.370 to -0.169)	**<0.0001**	0.007 (-0.080 to 0.095)	=0.8737	0.037 (-0.409 to -0.275)	=0.4126
TyG-WC	-0.243 (-0.342 to -0.138)	**<0.0001**	0.044 (-0.044 to 0.130)	=0.3307	0.040 (-0.456 to -0.328)	=0.3702
TyG-WHtR	-0.261 (-0.359 to -0.157)	**<0.0001**	0.051 (-0.037 to 0.137)	=0.2582	0.053 (-0.461 to -0.333)	=0.2379
lnTyG-HDL	-0.152 (-0.256 to -0.044)	**=0.0058**	-0.014 (-0.101 to 0.074)	=0.7561	0.002 (-0.314 to -0.171)	=0.9630
lnLAP	-0.203 (-0.304 to -0.096)	**=0.0002**	0.032 (-0.056 to 0.120)	=0.4707	0.022 (-0.471 to -0.344)	=0.6257
lnVAI	-0.144 (-0.248 to -0.036)	**=0.0092**	-0.006 (-0.094 to 0.082)	=0.8921	0.013 (-0.357 to -0.218)	=0.7715

All significant associations are shown in bold type. Confounders are age, regular alcohol intake, regular tobacco intake, treatment for hypertension, heart rate, and brachial pulse pressure. CI, confidence interval; E/A, early-to-late mitral velocity; e’, myocardial tissue lengthening in early diastole at the mitral annulus; E/e’,transmitral early blood flow velocity/velocity of the mean value of lateral and septal wall myocardial tissue lengthening in early diastole at the mitral annulus; EF, ejection fraction; GFR, estimated glomerular filtration rate; lnHOMA-IR, natural logarithm of homeostatic model assessment for insulin resistance; lnLAP, natural logarithm of lipid accumulation product; LVMI, left ventricular mass indexed to body surface area; LV RWT, left ventricular relative wall thickness; METS-IR, metabolic score for insulin resistance; mFS, left ventricular midwall fractional shortening; PWV, pulse wave velocity; QUICKI, quantitative insulin sensitivity check index; r, correlation coefficient; TyG, triglyceride–glucose index; TyG-BMI, triglyceride–body mass index; lnTyG-HDL, natural logarithm of triglycerides to high-density cholesterol concentrations; TyG-WC, triglyceride–waist circumference; TyG-WHtR, triglyceride–waist-to-height ratio; lnVAI, natural logarithm of visceral adiposity index.

Table S8. Relationships between various indices of insulin resistance and end-organ measures, independent of confounders, in the male participants (n=271).

	Partial r (95%CI)	p-value	Partial r (95%CI)	p-value	Partial r (95%CI)	p-value
	**LVMI**		**LV RWT**		**EF**	
lnHOMA-IR	0.152 (0.032 to 0.267)	**=0.0132**	0.216 (0.098 to 0.328)	**=0.0004**	-0.032 (-0.183 to 0.119)	=0.6754
QUICKI	-0.133 (-0.249 to -0.012)	**=0.0305**	-0.189 (-0.303 to -0.070)	**=0.0019**	0.044 (-0.108 to 0.194)	=0.5728
METS-IR	0.089 (-0.032 to 0.208)	=0.1465	0.131 (0.011 to 0.248)	**=0.0324**	-0.038 (-0.188 to 0.114)	=0.6254
TyG	0.023 (-0.098 to 0.143)	=0.7106	0.084 (-0.037 to 0.202)	=0.1736	-0.009 (-0.160 to 0.143)	=0.9090
TyG-BMI	0.094 (-0.027 to 0.212)	=0.1288	0.134 (0.014 to 0.251)	**=0.0286**	-0.040 (-0.190 to 0.112)	=0.6088
TyG-WC	0.090 (-0.031 to 0.208)	=0.1433	0.113 (-0.007 to 0.231)	=0.0653	-0.048 (-0.198 to 0.104)	=0.5343
TyG-WHtR	0.107 (-0.014 to 0.224)	=0.0833	0.129 (0.009 to 0.246)	**=0.0352**	-0.029 (-0.179 to 0.123)	=0.7100
lnTyG-HDL	0.016 (-0.104 to 0.136)	=0.7927	0.044 (-0.077 to 0.164)	=0.4750	0.052 (-0.100 to 0.202)	=0.5027
lnLAP	0.044 (-0.078 to 0.166)	=0.4777	0.066 (-0.056 to 0.187)	=0.2878	-0.012 (-0.164 to 0.139)	=0.8716
lnVAI	0.022 (-0.100 to 0.144)	=0.7204	0.042 (-0.081 to 0.163)	=0.5044	0.048 (-0.104 to 0.198)	=0.5340
	**mFS**		**E/A**		**E/e' (n=180)**	
lnHOMA-IR	-0.096 (-0.214 to 0.025)	=0.1202	-0.038 (-0.157 to 0.083)	=0.5432	0.249 (0.101 to 0.386)	=**0.0011**
QUICKI	0.069 (-0.052 to 0.188)	=0.2628	0.016 (-0.105 to 0.136)	=0.7976	-0.235 (-0.372 to -0.086)	=**0.0021**
METS-IR	-0.007 (-0.128 to 0.113)	=0.9081	-0.087 (-0.206 to 0.034)	=0.1558	0.196 (0.046 to 0.337)	**=0.0106**
TyG	-0.010 (-0.130 to 0.111)	=0.8749	-0.088 (-0.206 to 0.033)	=0.1550	0.139 (-0.013 to 0.284)	=0.0721
TyG-BMI	-0.040 (-0.159 to 0.081)	=0.5217	-0.121 (-0.238 to -0.001)	**=0.0491**	0.220 (0.070 to 0.359)	**=0.0041**
TyG-WC	-0.042 (-0.161 to 0.079)	=0.4981	-0.146 (-0.262 to -0.026)	**=0.0174**	0.182 (0.031 to 0.324)	**=0.0177**
TyG-WHtR	-0.030 (-0.150 to 0.091)	=0.6243	-0.154 (-0.269 to -0.034)	**=0.0119**	0.239 (0.090 to 0.376)	**=0.0017**
lnTyG-HDL	0.092 (-0.029 to 0.210)	=0.1365	-0.040 (-0.160 to 0.081)	=0.5132	0.080 (-0.072 to 0.228)	=0.3032
lnLAP	0.010 (-0.112 to 0.132)	=0.8758	-0.109 (-0.228 to 0.013)	=0.0802	0.138 (-0.014 to 0.283)	=0.0738
lnVAI	0.083 (-0.040 to 0.203)	=0.1841	-0.047 (-0.168 to 0.076)	=0.4575	0.079 (-0.073 to 0.228)	=0.3063
	**e' (n=180)**		**PWV**		**GFR**	
lnHOMA-IR	-0.206 (-0.346 to -0.056)	**=0.0072**	-0.042 (-0.162 to 0.079)	=0.4915	-0.205 (-0.317 to -0.086)	=**0.0008**
QUICKI	0.171 (0.019 to 0.314)	**=0.0267**	0.052 (-0.069 to 0.172)	=0.3975	0.208 (0.089 to 0.320)	=**0.0006**

METS-IR	-0.190 (-0.332 to -0.040)	**=0.0132**	-0.032 (-0.152 to 0.089)	=0.6062	-0.122 (-0.239 to -0.002)	**=0.0464**
TyG	-0.178 (-0.320 to -0.026)	**=0.0210**	0.001 (-0.120 to 0.121)	=0.9969	-0.053 (-0.172 to 0.069)	=0.3951
TyG-BMI	-0.243 (-0.379 to -0.094)	**=0.0015**	-0.015 (-0.135 to 0.106)	=0.8125	-0.129 (-0.246 to -0.008)	**=0.0356**
TyG-WC	-0.200 (-0.341 to -0.050)	**=0.0090**	0.010 (-0.110 to 0.131)	=0.8661	-0.110 (-0.227 to 0.011)	=0.0734
TyG-WHtR	-0.243 (-0.379 to -0.094)	**=0.0015**	0.007 (-0.114 to 0.127)	=0.9093	-0.103 (-0.220 to 0.018)	=0.0951
lnTyG-HDL	-0.120 (-0.266 to 0.033)	=0.1222	-0.047 (-0.167 to 0.074)	=0.4420	-0.032 (-0.152 to 0.089)	=0.6052
lnLAP	-0.206 (-0.346 to -0.056)	**=0.0071**	0.003 (-0.119 to 0.125)	=0.9644	-0.120 (-0.238 to 0.003)	=0.0549
lnVAI	-0.116 (-0.263 to 0.036)	=0.1339	-0.042 (-0.163 to 0.080)	=0.5005	-0.032 (-0.153 to 0.091)	=0.6107

All significant associations are shown in bold type. Confounders are age, regular alcohol intake, regular tobacco intake, treatment for hypertension, heart rate, and brachial pulse pressure. CI, confidence interval; E/A, early-to-late mitral velocity; e', myocardial tissue lengthening in early diastole at the mitral annulus; E/e', transmitral early blood flow velocity/velocity of the mean value of lateral and septal wall myocardial tissue lengthening in early diastole at the mitral annulus; EF, ejection fraction; GFR, estimated glomerular filtration rate; lnHOMA-IR, natural logarithm of homeostatic model assessment for insulin resistance; lnLAP, natural logarithm of lipid accumulation product; LVMI, left ventricular mass indexed to body surface area; LV RWT, left ventricular relative wall thickness; METS-IR, metabolic score for insulin resistance; mFS, left ventricular midwall fractional shortening; PWV, pulse wave velocity; QUICKI, quantitative insulin sensitivity check index; r, correlation coefficient; TyG, triglyceride–glucose index; TyG-BMI, triglyceride–body mass index; lnTyG-HDL, natural logarithm of triglycerides to high-density cholesterol concentrations; TyG-WC, triglyceride-waist circumference; TyG-WHtR, triglyceride–waist-to-height ratio; lnVAI, natural logarithm of visceral adiposity index.

Table S9. Wald X^2 for HOMA-IR or QUICKI compared to measures of adiposity (BMI and waist circumference), triglycerides, HDL cholesterol, and all other significant determinants of end-organ damage in the participants without DM (n=669).

	Wald X^2	p-value	Wald X^2	p-value	Wald X^2	p-value
	Model 1		Model 2		Model 3	
LVH-BSA vs.						
HOMA-IR	8.71	**=0.0032**	9.61	**=0.0019**	9.60	**=0.0019**
Age	9.69	**=0.0018**	11.62	**=0.0007**	12.03	**=0.0005**
BP*	13.16	**=0.0003**	11.44	**=0.0007**	11.52	**=0.0007**
HR	8.13	**=0.0043**	7.41	**=0.0065**	7.58	**=0.0059**
WC	—	—	-0.76	=0.3841	—	—
BMI	—	—	—	—	-1.24	=0.2662
Triglycerides	—	—	-2.52	=0.1124	-2.69	=0.1006
HDL cholesterol	—	—	-0.31	=0.5783	-0.35	=0.5557
LVH-BSA vs.						
QUICKI	-9.89	**=0.0017**	-11.01	**=0.0009**	-11.25	**=0.0008**
Age	10.55	**=0.0012**	12.67	**=0.0004**	13.17	**=0.0003**
BP*	15.24	**<0.0001**	13.72	**=0.0002**	13.85	**=0.0002**
HR	-8.03	**=0.0046**	-7.13	**=0.0076**	-7.32	**=0.0068**
WC	—	—	-1.00	=0.3161	—	—
BMI	—	—	—	—	-1.73	=0.1889
Triglycerides	—	—	-2.52	=0.1124	-2.75	=0.0971
HDL cholesterol	—	—	-0.15	=0.0983	-0.19	=0.6643
Concentricity vs.						
HOMA-IR	4.80	**=0.0284**	5.17	**=0.0230**	5.27	**=0.0217**
Age	5.37	**=0.0205**	5.74	**=0.0166**	6.52	**=0.0107**
WC	—	—	0.001	=0.9835	—	—
BMI	—	—	—	—	-0.06	=0.8033
Triglycerides	—	—	-1.39	=0.2386	-1.37	=0.2410
HDL cholesterol	—	—	-0.32	=0.5743	-0.37	=0.5436

Concentricity vs.						
QUICKI	-3.68	=0.0549	-3.81	=0.0508	-3.97	**=0.0464**
Age	5.89	**=0.0152**	6.15	**=0.0131**	7.00	**=0.0081**
WC	—	—	-0.002	=0.9621	—	—
BMI	—	—	—	—	-0.13	=0.7189
Triglycerides	—	—	-1.14	=0.2855	-1.13	=0.2867
HDL cholesterol	—	—	-0.21	=0.6500	-0.25	=0.6158
Incr PWV						
HOMA-IR	0.88	=0.3468	0.11	=0.7448	0.16	=0.6929
Age	49.53	**<0.0001**	42.47	**<0.0001**	45.20	**<0.0001**
BP*	14.43	**=0.0001**	10.10	**=0.0015**	10.06	**=0.0015**
HR	4.09	**=0.0431**	2.81	=0.0935	2.88	=0.0898
WC	—	—	0.21	=0.6428	—	—
BMI	—	—	—	—	0.006	=0.9376
Triglycerides	—	—	0.001	=0.9769	0.003	=0.9558
HDL cholesterol	—	—	-0.37	=0.5405	-0.46	=0.4987
Incr PWV						
QUICKI	-0.60	=0.4376	-0.03	=0.8712	-0.06	=0.8112
Age	49.86	**<0.0001**	42.45	**<0.0001**	45.28	**<0.0001**
BP*	14.41	**=0.0001**	9.94	**=0.0016**	9.88	**=0.0017**
HR	4.03	**=0.0448**	2.81	=0.0936	2.87	=0.0901
WC	—	—	0.23	=0.6290	—	—
BMI	—	—	—	—	0.007	=0.9310
Triglycerides	—	—	0.002	=0.9616	0.006	=0.9368
HDL cholesterol	—	—	-0.37	=0.5407	-0.45	=0.5029
Decr GFR						
HOMA-IR	7.55	**=0.0060**	11.85	**=0.0006**	11.21	**=0.0008**
Age	36.52	**<0.0001**	46.12	**<0.0001**	44.37	**<0.0001**
WC	—	—	-3.72	=0.0537	—	—
BMI	—	—	—	—	1.72	=0.1900

Triglycerides	—	—	-3.19	=0.0740	-3.81	=0.0509
HDL cholesterol	—	—	0.02	=0.8748	0.08	=0.7768
Decr GFR						
QUICKI	-6.17	**=0.0130**	-10.29	**=0.0013**	-9.88	**=0.0017**
Age	37.74	**<0.0001**	47.04	**<0.0001**	45.32	**<0.0001**
WC	—	—	-4.20	**=0.0404**	—	—
BMI	—	—	—	—	-2.26	=0.1329
Triglycerides	—	—	-2.64	=0.1039	-3.29	=0.0697
HDL cholesterol	—	—	0.14	=0.7033	0.23	=0.6296

All significant associations are shown in bold type. Model 1: Adjustments for age, sex, regular alcohol intake, regular tobacco intake, treatment for hypertension, heart rate, and brachial pulse pressure. Model 2: Adjustments as for Model 1 plus adjustments for triglycerides, HDL cholesterol, brachial SBP and DBP (instead of PP), and waist circumference. Model 3: Adjustments as for Model 1 plus adjustments for triglycerides, HDL cholesterol, brachial SBP and DBP (instead of PP), and BMI. * BP in Model 1 is brachial pulse pressure, in Model 2 is brachial systolic BP, and in Model 3 is brachial systolic BP. BMI, body mass index; BP, blood pressure; CI, confidence interval; Concentricity, increased left ventricular relative wall thickness; Decr, decreased; GFR, estimated glomerular filtration rate; Incr, increased; HOMA-IR, homeostatic model assessment for insulin resistance; LVH-BSA, increased left ventricular mass indexed to body surface area; PWV, pulse wave velocity; QUICKI, quantitative insulin sensitivity check index; WC, waist circumference.

Table S10. Wald X^2 for HOMA-IR or QUICKI compared to measures of adiposity (BMI and waist circumference), triglycerides, HDL cholesterol, and all other significant determinants of end-organ damage in the normotensive (NT) participants and the hypertensive (HT) participants with controlled blood pressure (BP) (n=505).

	Model 1		Model 2		Model 3	
	Wald X^2	p-value	Wald X^2	p-value	Wald X^2	p-value
LVH-BSA vs.						
HOMA-IR	2.89	=0.0892	2.82	=0.0934	2.61	=0.1059
Age	3.30	=0.0693	6.40	**=0.0114**	5.54	**=0.0186**
BP*	7.02	**=0.0081**	14.95	**=0.0001**	15.56	**<0.0001**
HR	-4.77	**=0.0289**	-4.11	**=0.0425**	-4.96	**=0.0259**
WC	—	—	-6.82	**=0.0090**	—	—
BMI	—	—	—	—	-7.00	**=0.0082**
Triglycerides	—	—	-1.09	=0.2965	-1.43	=0.2316
HDL cholesterol	—	—	-0.34	=0.5626	-0.36	=0.5496
LVH-BSA vs.						
QUICKI	-2.31	=0.1285	-2.52	=0.1126	-2.56	=0.1095
Age	3.53	=0.0602	6.68	**=0.0097**	5.80	**=0.0160**
BP*	7.27	**=0.0070**	14.97	**=0.0001**	15.57	**<0.0001**
HR	-5.01	**=0.0252**	-4.23	**=0.0398**	-5.13	**=0.0235**
WC	—	—	-7.53	**=0.0061**	—	—
BMI	—	—	—	—	-7.83	**=0.0051**
Triglycerides	—	—	-0.95	=0.3294	-1.34	=0.2462
HDL cholesterol	—	—	-0.28	=0.5952	-0.32	=0.5741
Concentricity vs.						
HOMA-IR	5.64	**=0.0176**	4.84	**=0.0278**	4.75	**=0.0294**
Age	2.32	=0.1278	2.34	=0.1262	3.46	=0.0630
WC	—	—	-0.26	=0.6128	—	—
BMI	—	—	—	—	-3.12	=0.0774
Triglycerides	—	—	-0.12	=0.7307	-0.13	=0.7134

HDL cholesterol	—	—	-0.03	=0.8609	-0.20	=0.6545
Concentricity vs.						
QUICKI	-4.72	**=0.0298**	-4.09	**=0.0431**	-4.70	**=0.0302**
Age	2.55	=0.1103	2.74	=0.0977	3.95	**=0.0469**
WC	—	—	-0.48	=0.4892	—	—
BMI	—	—	—	—	-3.97	**=0.0464**
Triglycerides	—	—	-0.10	=0.7532	-0.14	=0.7094
HDL cholesterol	—	—	-0.01	=0.9126	-0.15	=0.6985
Incr PWV						
HOMA-IR	1.75	=0.1865	0.61	=0.4343	0.66	=0.4167
Age	28.52	**<0.0001**	24.57	**<0.0001**	24.92	**<0.0001**
BP*	2.51	=0.1132	3.71	=0.0542	3.74	=0.0531
HR	5.08	**=0.0242**	3.81	=0.0745	4.24	**=0.0395**
WC	—	—	1.96	=0.1614	—	—
BMI	—	—	—	—	0.19	=0.6642
Triglycerides	—	—	0.10	=0.7472	0.13	=0.7196
HDL cholesterol	—	—	-0.15	=0.7022	-0.35	=0.5522
Incr PWV						
QUICKI	-1.56	=0.2114	-0.21	=0.6443	-0.35	=0.5538
Age	28.99	**<0.0001**	24.93	**<0.0001**	28.29	**<0.0001**
BP*	2.68	=0.1017	3.84	**=0.0500**	3.88	**=0.0489**
HR	4.61	**=0.0317**	3.13	=0.0771	4.11	**=0.0426**
WC	—	—	1.82	=0.1776	—	—
BMI	—	—	—	—	0.13	=0.7186
Triglycerides	—	—	0.14	=0.7108	0.14	=0.7046
HDL cholesterol	—	—	-0.13	=0.7152	-0.32	=0.5753
Decr GFR						
HOMA-IR	10.58	**=0.0011**	16.91	**<0.0001**	16.41	**<0.0001**
Age	25.43	**<0.0001**	38.08	**<0.0001**	35.50	**<0.0001**
WC	—	—	-5.45	**=0.0195**	—	—

BMI	—	—	—	—	-2.42	=0.1199
Triglycerides	—	—	-8.05	**=0.0045**	-8.95	**=0.0028**
HDL cholesterol	—	—	-0.80	=0.3697	-0.48	=0.4901
Decr GFR						
QUICKI	-2.98	=0.0844	-6.97	**=0.0083**	-6.40	**=0.0114**
Age	26.01	**<0.0001**	36.84	**<0.0001**	34.22	**<0.0001**
WC	—	—	-6.61	**=0.0102**	—	—
BMI	—	—	—	—	-3.41	=0.0650
Triglycerides	—	—	-5.48	**=0.0193**	-6.27	**=0.0123**
HDL cholesterol	—	—	-0.52	=0.4694	-0.27	=0.6027

All significant associations are shown in bold type. Model 1: Adjustments for age, sex, regular alcohol intake, regular tobacco intake, treatment for hypertension, heart rate, and brachial pulse pressure. Model 2: Adjustments as for Model 1 plus adjustments for triglycerides, HDL cholesterol, brachial SBP and DBP (instead of PP), and waist circumference. Model 3: Adjustments as for Model 1 plus adjustments for triglycerides, HDL cholesterol, brachial SBP and DBP (instead of PP), and BMI. * BP in Model 1 is brachial pulse pressure, in Model 2 is brachial systolic BP, and in Model 3 is brachial systolic BP. BMI, body mass index; BP, blood pressure; CI, confidence interval; Concentricity, increased left ventricular relative wall thickness; Decr, decreased; GFR, estimated glomerular filtration rate; Incr, increased; HOMA-IR, homeostatic model assessment for insulin resistance; LVH-BSA, increased left ventricular mass indexed to body surface area; PWV, pulse wave velocity; QUICKI, quantitative insulin sensitivity check index; WC, waist circumference.
